# Establishing the Thermodynamic Cards of Dipine Models’ Oxidative Metabolism on 21 Potential Elementary Steps

**DOI:** 10.3390/molecules29153706

**Published:** 2024-08-05

**Authors:** Guang-Bin Shen, Shun-Hang Gao, Yan-Wei Jia, Xiao-Qing Zhu, Bao-Chen Qian

**Affiliations:** 1 College of Medical Engineering, Jining Medical University, Jining 272000, China; gbshen@mail.jnmc.edu.cn (G.-B.S.); 18958699419@163.com (S.-H.G.); jia13953094699@163.com (Y.-W.J.); 2 The State Key Laboratory of Elemento-Organic Chemistry, Department of Chemistry, Nankai University, Tianjin 300071, China

**Keywords:** thermodynamic cards, dipine oxidative metabolism, elementary steps

## Abstract

Dipines are a type of important antihypertensive drug as L-calcium channel blockers, whose core skeleton is the 1,4-dihydropyridine structure. Since the dihydropyridine ring is a key structural factor for biological activity, the thermodynamics of the aromatization dihydropyridine ring is a significant feature parameter for understanding the mechanism and pathways of dipine metabolism in vivo. Herein, 4-substituted-phenyl-2,6-dimethyl-3,5-diethyl-formate-1,4-dihydropyridines are refined as the structurally closest dipine models to investigate the thermodynamic potential of dipine oxidative metabolism. In this work, the thermodynamic cards of dipine models’ aromatization on 21 potential elementary steps in acetonitrile have been established. Based on the thermodynamic cards, the thermodynamic properties of dipine models and related intermediates acting as electrons, hydrides, hydrogen atoms, protons, and two hydrogen ions (atoms) donors are discussed. Moreover, the thermodynamic cards are applied to evaluate the redox properties, and judge or reveal the possible oxidative mechanism of dipine models.

## 1. Introduction

**Figure 1:**
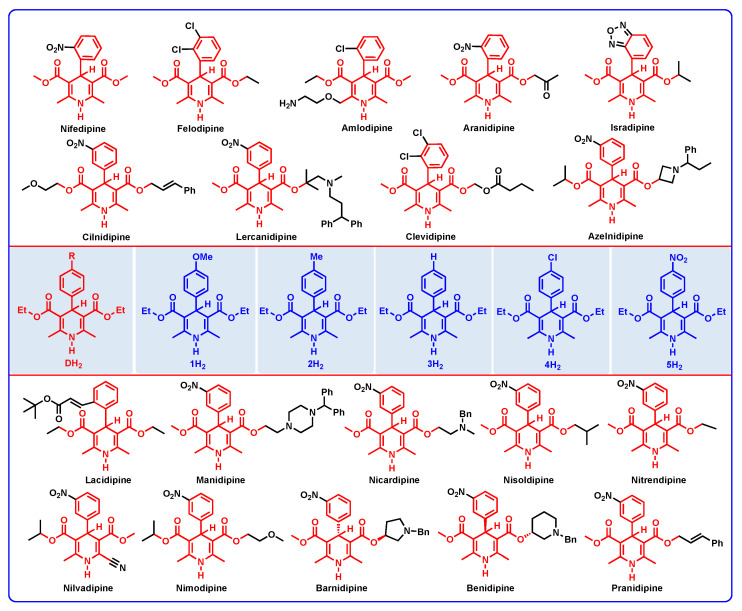
The chemical structures of dipines and refined dipine models (DH_2_).

**Figure 2:**
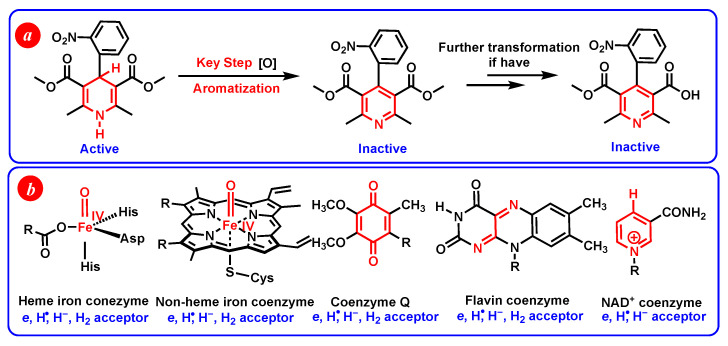
(**a**) The oxidative metabolism process of Nifedipine in vivo. (**b**) Some common oxidoreductases in vivo.

**Figure 3:**
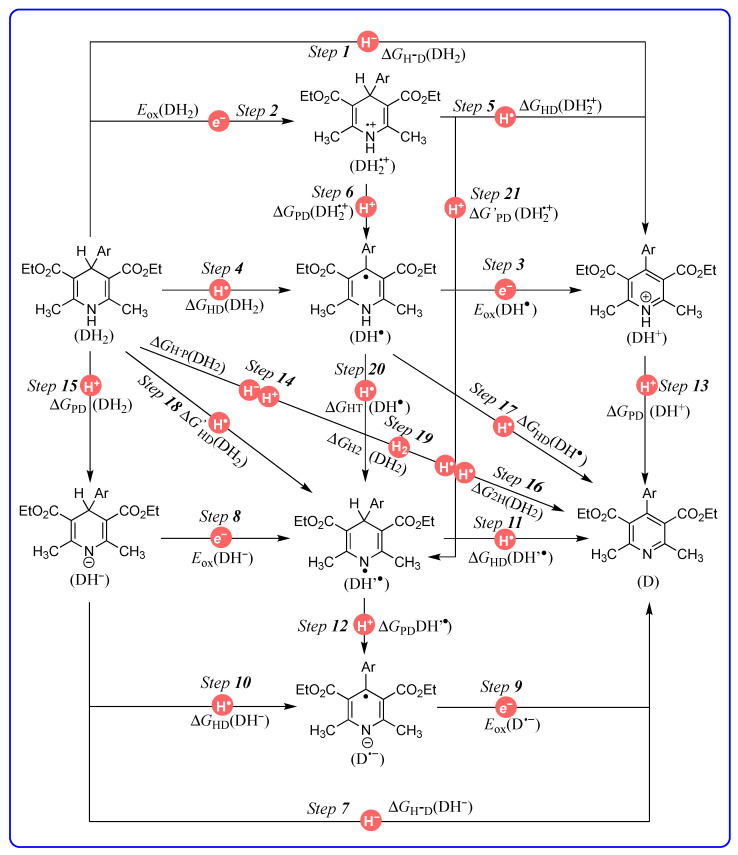
Thermodynamic cards of dipine model (DH_2_) aromatization on 21 potential elementary steps during oxidative metabolism.

1,4-dihydropyridines are a type of important antihypertensive drug known as L-calcium channel blockers [1,2,3,4,5,6]. Until now, over a dozen 1,4-dihydropyridine drugs have been extensively used in clinic, such as Nifedipine, Felodipine, Amlodipine, etc. (Figure 1) [7,8]. Because their drug names all contain the word “dipine”, they are also well-known as dipine drugs or dipines. The core skeleton of dipines is the 1,4-dihydropyridine structure, and the dihydropyridine ring is a key structural factor for their biological activity [7,8,9,10]. If the dihydropyridine ring is oxidized or aromatized into a pyridine structure in vivo, the resulting oxidative metabolites would lose the biological activity of blocking the L-calcium ion channel to lower blood pressure (Figure 2a) [1,2,3,4,5,6,7,8,9,10]. There exists various oxidoreductases in vivo (Figure 2b), such as CYP450 [11,12], flavin coenzyme [13,14,15], heme iron coenzyme [16,17,18,19], and coenzyme Q [20,21,22,23], as well as NADH coenzyme [24,25,26], and they are excellent electron, hydrogen atom, or hydride acceptors [27,28,29,30,31]. For example, it was reported that dipines were oxidated by cytochrome P-450 in human liver microsomes, and the metabolism process experienced a successive e + H^+^ + e + H^+^ mechanism [30]. Some highly active intermediates, including a radical cation, a radical, and a protonated pyridine, were involved in the oxidative process [30]. Therefore, the aromatized metabolism process of dipines may involve 21 possible thermodynamic elementary steps and six potential active intermediates (Figure 3), whose thermodynamic driving forces are the significant physical parameters for understanding and revealing the possible oxidative pathways of dipine metabolism in vivo.

From the view of structure, dipines are the analogues of the Hantzsch ester (YH_2_) [31] with the same core structure of 1,4-dihydropyridine, so dipines and the Hantzsch ester (YH_2_) all belong to NADH models. Further examining the structural characteristics of dipines in Figure 1, it can be discovered that they generally have the same parent structure of 4-aromatic-2,6-dimethyl-3,5-diformate-1,4-dihydropyridines. In this work, 4-substituted-phenyl-2,6-dimethyl-3,5-diethyl-formate-1,4-dihydropyridines (DH_2_) are refined as the structurally closest dipine models and represented all of the dipines to investigate the thermodynamic parameters of dipine oxidative metabolism (Figure 1).

Our group has long been committed to thermodynamic research on hydrogen transfer for NADH models, and determining the thermodynamic driving forces of over 200 organic hydrides releasing hydrides in non-aqueous media [27,31]. The previous works inspire us to further investigate and clarify the thermodynamic parameters of dipine aromatization in solution. Herein, the thermodynamic cards of dipine models’ aromatization on 21 potential elementary steps has been established (Figure 3). From the thermodynamic cards, the thermodynamic properties of dipine models and related intermediates acting as electrons, hydrides, hydrogen atoms, protons, and two hydrogen ions (atoms) donors are discussed. Furthermore, the thermodynamic cards are utilized to measure the redox properties, and diagnose the possible oxidative mechanism of dipine models.

## 2. Results and Discussion

### 2.1. Thermodynamic Parameters and Results

The thermodynamics of five 4-substituted-phenyl-2,6-dimethyl-3,5-diethyl-formate-1,4-dihydropyridines (DH_2_), including 4-(4-methoxyphenyl)-2,6-dimethyl-3,5-di(ethylformate)-1,4-dihydropyridine (1H_2_), 4-(4-methylphenyl)-2,6-dimethyl-3,5-di(ethylformate)-1,4-dihydropyridine (2H_2_), 4-phenyl-2,6-dimethyl-3,5-di(ethylformate)-1,4-dihydropyridine (3H_2_), 4-(4-chlorophenyl)-2,6-dimethyl-3,5-di(ethylformate)-1,4-dihydropyridine (4H_2_), and 4-(4-nitrophenyl)-2,6-dimethyl-3,5-di(ethylformate)-1,4-dihydropyridine (5H_2_) in acetonitrile were investigated, along with the thermodynamics of the Hantzsch ester (YH_2_) for better comparison.

The thermodynamic potentials of 21 elementary steps are defined as the corresponding Gibbs free energies for *Step 1*, *Steps 4–7*, *Steps 10–21* or potentials for *Step 2*, *Step 3*, *Step 8*, and *Step 9*, which are shown in Figure 3.

As can be seen from Figure 3, *Step 1* and *Step 7* are the hydride-releasing chemical processes, DH_2_ → DH^+^ + H^−^ and DH^−^ → D + H^−^, and their thermodynamic potentials are described by the related Gibbs free energies of DH_2_ and DH^−^ releasing hydrides, respectively, Δ*G*_H_^−^_D_(DH_2_) and Δ*G*_H_^−^_D_(DH^−^), which are also called hydricities [32,33,34]. Since the entropy change (TΔ*S*) of organic hydrides releasing hydrides is estimated as 4.9 kcal/mol [35] in acetonitrile, so the Δ*G*_H_^−^_D_(DH_2_) and Δ*G*_H_^−^_D_(DH^−^) are derived from their corresponding enthalpy changes, Δ*H*_H_^−^_D_(DH_2_) and Δ*H*_H_^−^_D_(DH^−^), by Equations (1) and (5) in Table 1. The reliability was proved in previous literature [36,37]. Δ*H*_H_^−^_D_(DH_2_) and Δ*H*_H_^−^_D_(DH^−^) were available from our previous work [27,38].

*Steps 2–3* and *Steps 8–9* are the electron-releasing chemical processes, and their thermodynamic potentials are described by the related oxidation potentials, *E*_ox_(DH_2_) for *Step 2*, *E*_ox_(DH^•^) for *Step 3*, *E*_ox_(DH^−^) for *Step 8*, and *E*_ox_(D^•−^) for *Step 9,* respectively. The oxidation potentials were determined in our previous work [27,38].

*Step 4–5*, *Steps 1–11,* and *Steps 17–18* are the hydrogen-atom-releasing chemical processes, and their thermodynamic potentials are described by the related Gibbs free energies, Δ*G*_HD_(DH_2_) for *Step 4*, Δ*G*_HD_(DH_2_^•+^) for *Step 5*, Δ*G*_HD_(DH^−^) for *Step 10*, Δ*G*_HD_(DH’^•^) for *Step 11*, Δ*G*_HD_(DH^•^) for *Step 17*, and Δ*G’*_HD_(DH_2_) for *Step 18,* respectively. Based on Hess’ law [27,37,38], Δ*G*_HD_(DH_2_), Δ*G*_HD_(DH_2_^•+^), Δ*G*_HD_(DH^−^), Δ*G*_HD_(DH’^•^), Δ*G*_HD_(DH^•^), and Δ*G’*_HD_(DH_2_) were calculated using Equations (2)–(3), (6)–(7), and (13)–(14), respectively, by constructing corresponding thermodynamic cycles, cycle *Step 4*–*Step 3*–*Step 1* for Δ*G*_HD_(DH_2_), cycle *Step 1*–*Step 2*–*Step 5* for Δ*G*_HD_(DH_2_^•+^), cycle *Step 10*–*Step 9*–*Step 7* for Δ*G*_HD_(DH^−^), cycle *Step 7*–*Step 8*–*Step 11* for Δ*G*_HD_(DH’^•^), cycle *Step 4*–*Step 17*–*Step 16* for Δ*G*_HD_(DH^•^), and cycle *Step 18*–*Step 11*–*Step 16* for Δ*G’*_HD_(DH_2_).

*Step 6*, *Steps 12–13, Step 15,* and *Step 21* are the proton-releasing chemical processes, and their thermodynamic potentials are described by the related Gibbs free energies, Δ*G*_PD_(DH_2_^•+^) for *Step 6*, Δ*G*_PD_(DH’^•^) for *Step 12*, Δ*G*_PD_(DH^+^) for *Step 13*, Δ*G*_PD_(DH_2_) for *Step 15*, and Δ*G’*_PD_(DH_2_^•+^) for *Step 21,* respectively. Δ*G*_PD_(DH^+^) for *Step 13* could be calculated by Equation (10) using the p*K*_a_(DH^+^) value, Δ*G*_PD_(DH^+^) = 1.37p*K*_a_(DH^+^) [39]. In this work, the p*K*_a_(DH^+^) values were predicted using the method developed by Luo and coworker at 2020 [40]. Based on Hess’ law [27,37,38], the Δ*G*_PD_(DH_2_^•+^), Δ*G*_PD_(DH’^•^), Δ*G*_PD_(DH_2_), and Δ*G’*_PD_(DH_2_^•+^) were calculated using Equations (4), (8), (11), and (17) in Table 1, respectively, by constructing corresponding thermodynamic cycles, cycle *Step 4*–*Step 2*–*Step 6* for Δ*G*_PD_(DH_2_^•+^), cycle *Step 8*–*Step 12*–*Step 10* for Δ*G*_PD_(DH’^•^), cycle *Step 14*–*Step 15*–*Step 7* for Δ*G*_PD_(DH_2_), and cycle *Step 6*–*Step 20*–*Step 21* for Δ*G’*_PD_(DH_2_^•+^).

*Step 14* is the chemical process of DH_2_ releasing two hydrogen ions (H^−^ + H^+^), and the thermodynamic potential is described by the Gibbs free energy of DH_2_ releasing two hydrogen ions, Δ*G*_H_^−^_P_(DH_2_). Δ*G*_H_^−^_P_(DH_2_) could be obtained from Δ*G*_H_^−^_D_(DH_2_) and Δ*G*_PD_(DH^+^) via Equation (10) in Table 1, Δ*G*_H_^−^_P_(DH_2_) = Δ*G*_H_-_D_(DH_2_) + Δ*G*_PD_(DH^+^) (Equation (10)), by constructing the thermodynamic cycle [27] *Step 1*–*Step 13*–*Step 14*.

*Step 16* is the chemical process of DH_2_ releasing two hydrogen atoms, and the thermodynamic potential is described by the Gibbs free energy of DH_2_ releasing two hydrogen atoms, Δ*G*_2H_(DH_2_). Δ*G*_2H_(DH_2_) could be obtained by Equation (12) in Table 1, Δ*G*_2H_(DH_2_) = Δ*G*_H_^−^_P_(DH_2_) + *F*[*E*_red_(H^•^) − *E*_ox_(H^•^)] (Equation (12)). Among Equation (12), *E*_red_(H^•^) and *E*_ox_(H^•^) were reported as −1.137 V and −2.307 V (vs. Fc) [27,38] in acetonitrile.

*Step 19* is the chemical process of DH_2_ releasing hydrogen gas (H_2_), and the thermodynamic potential is described by the Gibbs free energy of DH_2_ releasing H_2_, Δ*G*_H2_(DH_2_). Δ*G*_H2_(DH_2_) could be obtained by Equation (15) in Table 1, Δ*G*_H2_(DH_2_) = Δ*G*_2H_(DH_2_) − Δ*G*_H_^−^_D_(H_2_) (Equation (15)). Among Equation (15), Δ*G*_H_^−^_D_(H_2_) refers to the Gibbs free energy of H_2_ releasing hydrides, which was reported as 76.0 kcal/mol [41,42] in acetonitrile.

*Step 20* is the chemical process of a hydrogen atom transfer within a molecule from the N_1_-position to the C_4_-position, and the thermodynamic potential is described by the related Gibbs free energy, Δ*G*_HT_(DH^•^). Δ*G*_HT_(DH^•^) could be obtained by Equation (16) in Table 1, Δ*G*_HT_(DH^•^) = Δ*G*_HD_(DH^•^) − Δ*G*_HD_(DH’^•^) (Equation (16)) by constructing the thermodynamic cycle [27] *Step 20*–*Step 11*–*Step 17*.

**Table 1:** Chemical processes, thermodynamic potentials, and computed equations or data sources of 21 elementary steps for DH_2_ aromatization.

*Step X*	Chemical Process	Potentials	Computed Equations or Data Sources	Equation
*Step 1*	DH_2_ → DH^+^ + H^−^	Δ*G*_H_^−^_D_(DH_2_)	Δ*G*_H_^−^_D_(DH_2_) = ^−^Δ*H*_H_^−^_D_(DH_2_) − 4.9	(1)
*Step 2*	DH_2_ → DH_2_^•+^ + e^−^	*E*_ox_(DH_2_)	[38]	-
*Step 3*	DH^•^ → DH^+^ + e^−^	*E*_ox_(DH^•^)	[38]	-
*Step 4*	DH_2_ → DH^•^ + H^•^	Δ*G*_HD_(DH_2_)	Δ*G*_HD_(DH_2_) = Δ*G*_H_^−^_D_(DH_2_) − *F*[*E*_red_(DH^+^) − *E*_ox_(H^−^)]	(2)
*Step 5*	DH_2_^•+^ → DH^+^ + H^•^	Δ*G*_HD_(DH_2_^•+^)	Δ*G*_HD_(DH_2_^•+^) = Δ*G*_H_^−^_D_(DH_2_) − *F*[*E*_ox_(DH_2_) − *E*_red_(H^•^)]	(3)
*Step 6*	DH_2_^•+^ → DH^•^ + H^+^	Δ*G*_PD_(DH_2_^•+^)	Δ*G*_PD_(DH_2_^•+^) = Δ*G*_HD_(DH_2_) − *F*[*E*_ox_(DH_2_) − *E*_red_(H^+^)]	(4)
*Step 7*	DH^−^ → D + H^−^	Δ*G*_H_^−^_D_(DH^−^)	Δ*G*_H_^−^_D_(DH^−^) = Δ*H*_H_^−^_D_(DH^−^) − 4.9	(5)
*Step 8*	DH^−^ → DH^•^ + e^−^	*E*_ox_(DH^−^)	[38]	-
*Step 9*	D^•−^ → D + e^−^	*E*_ox_(D^•−^)	[38]	-
*Step 10*	DH^−^ → D^•−^ + H^•^	Δ*G*_HD_(DH^−^)	Δ*G*_HD_(DH^−^) = Δ*G*_H_-_D_(DH^−^) − *F*[*E*_red_(D) − *E*_ox_(H^−^)]	(6)
*Step 11*	DH’^•^ → D + H^•^	Δ*G*_HD_(DH’^•^)	Δ*G*_HD_(DH’^•^) = Δ*G*_H_-_D_(DH^−^) − *F*[*E*_ox_(DH^−^) − *E*_red_(H^•^)]	(7)
*Step 12*	DH’^•^ → D^−^ + H^+^	Δ*G*_PD_(DH’^•^)	Δ*G*_PD_(DH’^•^) = Δ*G*_HD_(DH^−^) − *F*[*E*_ox_(DH^−^) − *E*_red_(H^+^)]	(8)
*Step 13*	DH^+^ → D + H^+^	Δ*G*_PD_(DH^+^)	Δ*G*_PD_(DH^+^) = 1.37 p*K*_a_(DH^+^)	(9)
*Step 14*	DH_2_ → D + H^−^ + H^+^	Δ*G*_H_^−^_P_(DH_2_)	Δ*G*_H_^−^_P_(DH_2_) = Δ*G*_H_-_D_(DH_2_) + Δ*G*_PD_(DH^+^)	(10)
*Step 15*	DH_2_ → DH^−^ + H^+^	Δ*G*_PD_(DH_2_)	Δ*G*_PD_(DH_2_) = Δ*G*_H_^−^_P_(DH_2_) − Δ*G*_H_^−^_D_(DH^−^)	(11)
*Step 16*	DH_2_ → D + H^•^ + H^•^	Δ*G*_2H_(DH_2_)	Δ*G*_2H_(DH_2_) = Δ*G*_H_^−^_P_(DH_2_) + *F*[*E*_red_(H^•^) − *E*_ox_(H^•^)]	(12)
*Step 17*	DH^•^ → D + H^•^	Δ*G*_HD_(DH^•^)	Δ*G*_HD_(DH^•^) = Δ*G*_2H_(DH_2_) − Δ*G*_HD_(DH_2_)	(13)
*Step 18*	DH_2_ → DH’^•^ + H^•^	Δ*G’*_HD_(DH_2_)	Δ*G*_HD_(DH_2_) = Δ*G*_2H_(DH_2_) − Δ*G*_HD_(DH’^•^)	(14)
*Step 19*	DH_2_ → D + H_2_	Δ*G*_H2_(DH_2_)	Δ*G*_H2_(DH_2_) = Δ*G*_2H_(DH_2_) − Δ*G*_H_^−^_D_(H_2_)	(15)
*Step 20*	DH^•^ → DH’^•^	Δ*G*_HT_(DH^•^)	Δ*G*_HT_(DH^•^) = Δ*G*_HD_(DH^•^) − Δ*G*_HD_(DH’^•^)	(16)
*Step 21*	DH_2_^•+^ → DH’^•^ + H^+^	Δ*G’*_PD_(DH_2_^•+^)	Δ*G’*_PD_(DH_2_^•+^) = Δ*G*_PD_(DH_2_^•+^) + Δ*G*_HT_(DH^•^)	(17)

Note: The unit of potentials is V vs. Fc. The unit of Gibbs free energies is kcal/mol. *E*_ox_(H^−^) = *E*_red_(H^•^) = −1.137 V vs. Fc in acetonitrile [38]. *E*_red_(H^+^) = *E*_ox_(H^•^) = −2.307 V vs. Fc in acetonitrile [38]. Δ*G*_H_^−^_D_(H_2_) = 76.0 kcal/mol in acetonitrile [41].

*Step 21* is the chemical process of DH_2_^•+^ releasing protons from the N_1_-position, and the thermodynamic potential is described by the related Gibbs free energy, Δ*G’*_PD_(DH_2_^•+^). Δ*G’*_PD_(DH_2_^•+^) could be obtained by Equation (17) in Table 1, Δ*G’*_PD_(DH_2_^•+^) = Δ*G*_PD_(DH_2_^•+^) + Δ*G*_HT_(DH^•^) (Equation (17)) by constructing the thermodynamic cycle [27] *Step 6*–*Step 20*–*Step 21*.

All of the chemical processes, thermodynamic potentials, computed equations, or data sources of the 21 elementary steps for DH_2_ aromatization are listed in Table 1. Moreover, the thermodynamic results of 1H_2_–5H_2_ and YH_2_ aromatization are shown in Table 2. Accordingly, the thermodynamic cards [27,31,38] of 1H_2_–5H_2_ (Figures S1–S5) and YH_2_ (Figure S6) on 21 potential elementary steps are established and presented in the Supporting Information to make them more convenient to query and use. Obviously, the thermodynamic cards (Figure 3 and Figures S1–S5) can visually exhibit the mutual transformations among dipine models DH_2_, aromatic products D, and the related six active intermediates, as well as the thermodynamic driving forces of the related 21 potential elementary steps. Naturally, the thermodynamic cards could be employed to quantitatively measure and predict the characteristic chemical or thermodynamic properties of the dipine models and involved intermediates.

### 2.2. The Acidities of DH^+^ in Acetonitrile

**Figure 4:**
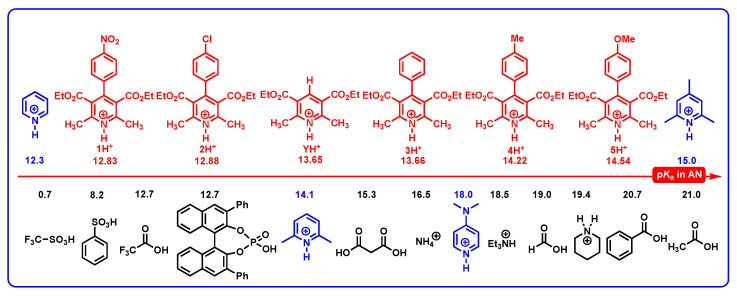
The p*K*_a_ of DH^+^ and YH^+^, along with the p*K*_a_ of common organic acids in acetonitrile.

In our previous work [39], the p*K*_a_ of 78 protonated pyridines in acetonitrile were predicted using the method developed by Luo and coworker at 2020 [40], and the predicted accuracy was estimated as ±1 p*K*_a_. Herein, the p*K*_a_ of DH^+^ and YH^+^ with typical structures of protonated pyridine in acetonitrile are also predicted using the same method. To better understand the acidities of DH^+^ and YH^+^ in acetonitrile, the p*K*_a_ values of DH^+^ and YH^+^, along with the p*K*_a_ of common organic acids in acetonitrile, are displayed in Figure 4. From Figure 4, it is found that the p*K*_a_(DH^+^) scale ranges from 12.83 for 1H^+^ to 14.54 for 5H^+^, and the acidities of DH^+^ (12.83–14.54) are between PyH^+^ (12.3) [43,44,45] and protonated 2,4,6-trimethylpyridine (15.0) [43,44,45], which further verify the accuracy of the predicted p*K*_a_(DH^+^) values.

What is more, several interesting conclusions could be drawn from Figure 4. (1) Since the p*K*_a_ of BINOL-derived phosphoric acids (BPA) are reported as 12–14 in MeCN [46,47,48,49], DH^+^ (12.83–14.54) have comparable acidities to BPA. (2) DH^+^ (12.83–14.54) generally belongs to medium–strong organic acids, which could be applied in acid-catalyzed chemical reactions. (3) The acidity of 3H^+^ (13.66) is almost equal to that of YH^+^ (13.65), and when the 4-phenyl group of DH^+^ is substituted by an electron-withdrawing group, the acidity of new DH^+^ is stronger than 3H^+^ (13.66) or YH^+^ (13.65). In contrast, if the 4-phenyl group of DH^+^ is substituted by an electron-donating group, the acidity of new DH^+^ is weaker than 3H^+^ (13.66) or YH^+^ (13.65). (4) Sometimes, after DH_2_ are oxidized by hydride acceptors, the acidities of resulting DH^+^ should be considered when the organic bases are involved in the reaction system. (5) D (p*K*_a_(DH^+^) = 12.83 − 14.54) could be used as alternative organic bases of pyridine (p*K*_a_ of conjugate acid is 12.3) [43,44,45], 2,6-trimethylpyridine (p*K*_a_ of conjugate acid is 14.1) [43,44,45], and 2,4,6-trimethylpyridine (p*K*_a_ of its conjugate acid is 15.0) [43,44,45] in acetonitrile.

### 2.3. Thermodynamic Properties of DH_2_ and Related Intermediates Acting as Electrons, Hydrides, Hydrogen Atoms, Protons, and Two Hydrogen Ions (Atoms) Donors in Acetonitrile

#### 2.3.1. Electron-Donating Properties

**Figure 5:**
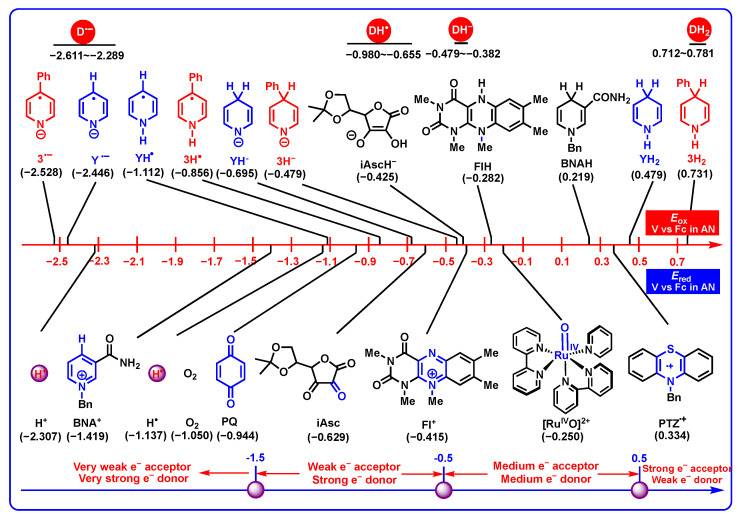
Oxidation potentials (*E*_ox_) of DH_2_, DH^−^, DH^•^, and D^•−^, as well as the reduction potentials (*E*_red_) of common coenzyme models and electron acceptors in acetonitrile (V vs. Fc).

To clearly compare the electron-donating properties of DH_2_ and related intermediates (DH^−^, DH^•^, and D^•−^), the oxidation potentials (*E*_ox_ vs. Fc) of DH_2_, DH^−^, DH^•^, and D^•−^, as well as the reduction potentials (*E*_red_ vs. Fc) of common coenzyme models (BNA^+^ for NADH coenzyme, PQ for coenzyme Q, Asc for oxidated ascorbate, Fl^+^ for flavin coenzyme, and Ru^IV^O^2+^ for heme enzyme) [40,42] and electron acceptors (H^+^, H^•^, O_2_, and PTZ^•+^) [29,50] in acetonitrile are exhibited in Figure 5. From Figure 5, it is clear that the oxidation potential scale is from 0.712 V to 0.781 V for DH_2_, from −0.382 V to −0.497 V for DH^−^, from −0.980 V to −0.655 V for DH^•^, and from −2.611 V to −2.289 V for D^•−^, indicating that DH^•−^ are the thermodynamically best electron donors, even better than H^•^ (*E*_ox_ = −2.307 V) [38], and DH_2_ are the thermodynamically worse electron donors than BNAH (0.219 V) [27]. The electron-donating abilities increase in the following order of DH_2_ < DH^−^ < DH^•^ < D^•−^. According to their thermodynamic range, DH_2_ (0.712–0.781 V), DH^−^ (−0.382–−0.497 V), DH^•^ (−0.980–−0.655 V), and D^•−^ (−2.611–−2.289 V) are recognized as the weak electron donors, medium–strong electron donors, strong electron donors, and very strong electron donors, respectively.

Since ascorbate is a good single-electron donor in vivo, the *E*_ox_(DH_2_) (0.712–0.781 V) is greater than 5,6-isopropylidene ascorbate (iAscH^−^, −0.425 V) [27], and the *E*_ox_(DH^•^) (−0.980–−0.655 V), *E*_ox_(DH^−^) (−0.382–−0.497 V), and *E*_ox_(D^•−^) (−2.611–−2.289 V) are more negative than iAscH^−^ (−0.425 V) [27], which means that DH_2_ are thermodynamically worse electron donors than iAscH^−^, and DH^•^, DH^−^, and D^•−^ are thermodynamically better electron donors than iAscH^−^. In addition, Ru^IV^O^2+^ is known as the model of high-valence metal oxides, such as heme or non-heme iron coenzyme [16,17,18,19], and cytochrome P450 [11,12,13,14,15]. The reduction potential of Ru^IV^O^2+^ is determined as −0.250 V (vs. Fc) [51] in acetonitrile; therefore, the electron transfers from DH^−^ (−0.382–−0.497 V), DH^•^ (−0.980–−0.655 V), and D^•−^ (−2.611–−2.289 V) to Ru^IV^O^2+^ are thermodynamically favorable with large thermodynamic driving forces, while the electron transfers from DH_2_ (0.71–0.781 V) to Ru^IV^O^2+^ are thermodynamically unfavorable with Δ*G*_ET_(DH_2_/Ru^IV^O^2+^) ≥ 22.2 kcal/mol.

It is well-known that O_2_ often functions as the electron acceptor in vivo, and the reduction potential of O_2_ was determined as −1.050 V (vs. Fc) [29] in acetonitrile. The thermodynamic analyses indicate that the electron transfers from DH^−^ (−0.382–−0.497 V), DH^•^ (−0.980–−0.655 V) and DH_2_ (0.712–0.781 V) to O_2_ are thermodynamically unfavorable, while electron transfers from D^•−^ (−2.611–−2.289 V) to O_2_ are thermodynamically feasible with Δ*G*_ET_(D^•−^/O_2_) ≤ −28.6 kcal/mol. Further considering the thermodynamic data, the Gibbs free energies of electron transfers from DH_2_ (0.712–0.781 V) to O_2_, DH_2_ + O_2_ → DH_2_^•+^ + O_2_^•−^, are estimated to be as high as 40.6–42.2 kcal/mol [Δ*G*_ET_(DH_2_/O_2_)], meaning that DH_2_ is stable in the air, and the direct electron transfer from DH_2_ to O_2_ is thermodynamically unfeasible. In contrast, the Gibbs free energies of electron transfers from DH^−^ (−0.832–−0.479 V) and DH^•^ (−0.980–−0.655 V) to O_2_, are calculated as 5.0–13.2 kcal/mol and 1.6–9.1 kcal/mol separately, which are slightly large energy barriers to surpass.

#### 2.3.2. Hydride-Donating Properties

**Figure 6:**
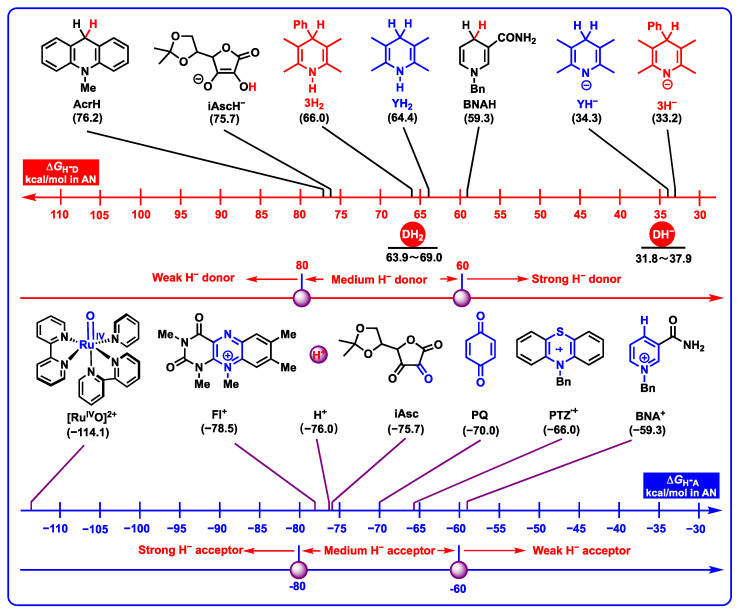
Hydricities of DH_2_, and DH^−^, as well as H^−^-affinities of common coenzyme models and hydride acceptors in acetonitrile (kcal/mol).

For a better comparison, the hydride-donating properties of DH_2_ and DH^−^ and the hydricities of DH_2_, DH^−^, and common hydride donors (iAscH^−^, BNAH, and AcrH), as well as the hydride-affinities of common coenzyme models (BNA^+^ for NADH coenzyme, PQ for coenzyme Q, iAsc for oxidated ascorbic acid, Fl^+^ for flavin coenzyme, and Ru^IV^O^2+^ for heme enzyme) and hydride acceptors (H^+^ and PTZ^•+^) in acetonitrile are displayed in Figure 6. As can be seen from Figure 6, it is found that the hydricity ranges from 63.9 to 69.0 kcal/mol for DH_2_, and from 31.8 to 37.9 kcal/mol for DH^−^. If the hydricities between DH_2_ and DH^−^ are compared, it is easy to discover that the hydricities of DH^−^ are greatly improved by the negative charge at the N_1_-atom compared with their parents DH_2_, and the hydride-donating abilities of DH^−^ (31.8–37.9 kcal/mol) are greater than DH_2_ (63.9–69.0 kcal/mol) by more than 30 kcal/mol. Based on their thermodynamic range, DH_2_ (63.9–69.0 kcal/mol) belong to thermodynamically medium–strong hydride donors, while DH^−^ (31.8–37.9 kcal/mol) belong to thermodynamically strong hydride donors, respectively.

DH_2_ are thermodynamically worse hydride donors than BNAH and thermodynamically better hydride donors than iAscH^−^, due to the hydricities of DH_2_ (63.9–69.0 kcal/mol) being more negative than BNAH (59.3 kcal/mol) [27] by 4.6–9.7 kcal/mol, and greater than iAscH^−^ (75.7 kcal/mol) [27] by 6.7–11.8 kcal/mol. DH^−^ are much better hydride donors than BNAH and iAscH^−^, and the hydricities of DH^−^ (31.8–37.9 kcal/mol) are much greater than BNAH (59.3 kcal/mol) and iAscH^−^ (75.7 kcal/mol) by more than 20 kcal/mol. The above thermodynamic data indicate that DH_2_ could not be oxidated by BNA^+^ through hydride transfer with related Gibbs free energies greater than 0, 4.6 kcal/mol ≤ Δ*G*_H_-_T_(DH_2_/BNA^+^) ≤ 9.7 kcal/mol, but the anion intermediates of DH_2_ (DH^−^) could be oxidated by BNA^+^ through hydride transfer with Gibbs free energies less than 0, −27.5 kcal/mol ≤ Δ*G*_H_-_T_(DH^−^/BNA^+^) ≤ −21.4 kcal/mol. In addition, DH_2_ and DH^−^ could be oxidated by iAsc (−75.7 kcal/mol) by hydride transfer with Gibbs free energies less than 0, −9.7 kcal/mol ≤ Δ*G*_H_-_T_(DH_2_/iAsc) ≤ −4.6 kcal/mol and −43.9 kcal/mol ≤ Δ*G*_H_-_T_(DH^−^/iAsc) ≤ −37.8 kcal/mol. What is more, since all of the Gibbs free energies of hydride transfer processes from DH_2_ to Ru^IV^O^2+^ (−114.1 kcal/mol), from DH_2_ to Fl^+^ (−78.5 kcal/mol), from DH_2_ to PQ (−70.0 kcal/mol), and from DH_2_ to H^+^ (−76.0 kcal/mol) are less than 0, it can be inferred that dipines may be oxidated by heme enzyme, cytochrome P450, flavin coenzyme, coenzyme Q, and H^+^ under suitable oxidoreductase by hydride oxidation in vivo.

#### 2.3.3. Hydrogen-Atom-Donating Properties

**Figure 7:**
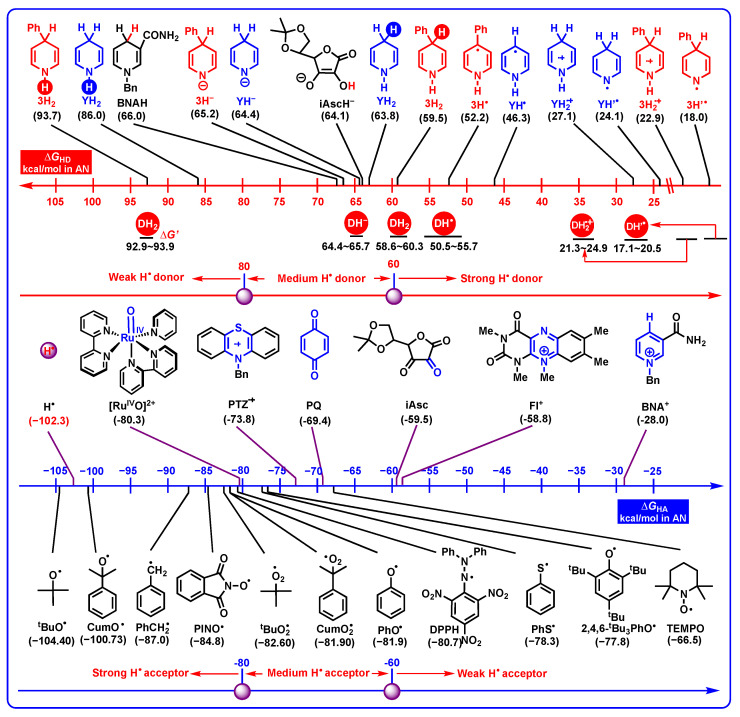
Thermodynamic hydrogen-atom-donating abilities of DH_2_, DH^−^, DH_2_^•+^, DH^•^, and DH’^•^, as well as hydrogen atom affinities of common radicals and coenzyme models in acetonitrile (kcal/mol).

Due to the N-H bond and C-H bond at the 1-position and 4-position of DH_2_, there are six possible elementary steps to release hydrogen atoms during the aromatization. Herein, the thermodynamic hydrogen-atom-donating abilities of DH_2_, DH^−^, DH_2_^•+^, DH^•^, and DH’^•^, as well as the hydrogen-atom affinities of 12 common radicals (involving H^•^, ^t^BuO^•^, CumO^•^, PhCH_2_^•^, PINO^•^, ^t^BuO_2_^•^, CumO_2_^•^, PhO^•^, DPPH, PhS^•^, 2,4,6-^t^Bu_3_PhO^•^, and TEMPO) [29] and coenzyme models (BNA^+^ for NADH coenzyme, PQ for coenzyme Q, iAsc for oxidated ascorbic acid, Fl^+^ for flavin coenzyme, and Ru^IV^O^2+^ for heme enzyme) in acetonitrile are shown in Figure 7.

From Figure 7, the thermodynamic hydrogen-atom-donating ability scale ranges from 92.9 to 93.9 kcal/mol for DH_2_ releasing hydrogen atoms from N_1_-H, from 58.6 to 60.3 kcal/mol for DH_2_ releasing hydrogen atoms from C_4_-H, from 64.4 to 65.7 kcal/mol for DH^−^, from 21.3 to 34.9 kcal/mol for DH_2_^•+^, from 50.5 to 55.7 kcal/mol for DH^•^, and from 17.1 to 20.5 kcal/mol for DH’^•^, and the thermodynamic hydrogen-atom-donating abilities increase in the order of DH_2_ (N_1_-H, 92.9–93.9 kcal/mol) < DH^−^ (64.4–65.7 kcal/mol) < DH_2_ (C_4_-H) (58.6–60.3 kcal/mol) < DH^•^ (50.5–55.7 kcal/mol) < DH_2_^•+^ (21.3–34.9 kcal/mol) < DH’^•^ (17.1–20.5 kcal/mol). According to their thermodynamic ranges, DH^•^, DH_2_^•+^, and DH’^•^ belong to thermodynamically strong hydrogen atom donors, DH^−^ and DH_2_ generally belong to thermodynamically medium–strong hydrogen atom donors to break C_4_-H bonds, while DH_2_ belong to thermodynamically weak hydrogen atom-donors to break N_1_-H bonds. Based on the above analysis, whether DH_2_ are medium–strong hydrogen atom donors or weak hydrogen atom donors depends on which hydrogen atoms DH_2_ releases, N_1_-H bonds or C_4_-H bonds. Generally, DH_2_ prefer to release hydrogen atoms from C_4_-H bonds (58.6–60.3 kcal/mol) instead of N_1_-H bonds (92.9–93.9 kcal/mol) from thermodynamics.

Since ascorbate is an excellent antioxidant to quench radicals in vivo [52], it is necessary to compare the thermodynamic properties of iAscH^−^, DH_2_, and related intermediates releasing hydrogen atoms. Several interesting conclusions could be made as follows. It is revealed that (1) the thermodynamic antioxidant potentials of DH^−^ (64.4–65.7 kcal/mol) are comparable to iAscH^−^ (64.1 kcal/mol) [27,52]. (2) DH_2_ (C_4_-H homolysis, 58.6–60.3 kcal/mol), DH^•^ (50.5–55.7 kcal/mol), DH_2_^•+^ (21.3–34.9 kcal/mol), and DH’^•^ (17.1–20.5 kcal/mol) are thermodynamically better antioxidants than iAscH^−^ (64.1 kcal/mol). (3) Unlike hydricities, the thermodynamic hydrogen-atom-donating abilities of DH^−^ decrease as a result of the negative charge at the N_1_-atom compared with their parents DH_2_, and the thermodynamic hydrogen-atom-donating abilities of DH^−^ (64.4–65.7 kcal/mol) are more negative than DH_2_ (58.6–60.3 kcal/mol) by 4.1–7.1 kcal/mol. (4) Because the final products of DH^•^ and DH’^•^ releasing hydrogen atoms are the same (D and H^•^), DH^•^ → D + H^•^ and DH’^•^ → D + H^•^. When the hydrogen-atom-donating Gibbs free energies of DH^•^ (50.5–55.7 kcal/mol) and DH’^•^ (17.1–20.5 kcal/mol) are compared, it can also be deduced that DH^•^ are more thermodynamically stable radicals than DH’^•^, and the Gibbs free energies of hydrogen atom transfer within DH^•^ from the N_1_-position to the C_4_-position [Δ*G*_HT_(DH^•^) for *Step 20* in Figure 3] are computed as 33.4–35.2 kcal/mol, which further verify the relative stability of DH^•^ and DH’^•^ in solution.

BNAH is the structurally closest model of the NADH coenzyme, and the hydrogen-atom-donating Gibbs free energy of BNAH is 66.0 kcal/mol [27]. It is clear that DH_2_ (C_4_-H homolysis, 58.6–60.3 kcal/mol), DH^•^ (50.5–55.7 kcal/mol), DH_2_^•+^ (21.3–34.9 kcal/mol), and DH’^•^ (17.1–20.5 kcal/mol) are thermodynamically better hydrogen atom donors than BNAH (66.0 kcal/mol), while DH_2_ are thermodynamically much weaker hydrogen atom donors than BNAH (66.0 kcal/mol) if the DH_2_ break N_1_-H bonds (92.9–93.9 kcal/mol).

As for the common 11 organic radicals collected in Figure 7, involving ^t^BuO^•^, CumO^•^, PhCH_2_^•^, PINO^•^, ^t^BuO_2_^•^, CumO_2_^•^, PhO^•^, DPPH, PhS^•^, 2,4,6-^t^Bu_3_PhO^•^, and TEMPO [29,53,54], the hydrogen atom affinity scale ranges from −66.5 for TEMPO to −104.40 kcal/mol for ^t^BuO^•^. All of the 11 radicals (−66.5–−104.40 kcal/mol) could thermodynamically oxidize DH^−^ (64.4–65.7 kcal/mol), DH_2_ (breaking C_4_-H) (58.6–60.3 kcal/mol), DH^•^ (50.5–55.7 kcal/mol), DH_2_^•+^ (21.3–34.9 kcal/mol), and DH’^•^ (17.1–20.5 kcal/mol) via hydrogen atoms transfer. Only ^t^BuO^•^ (−104.40 kcal/mol) and CumO^•^ (−110.73 kcal/mol) could oxidize DH_2_ by N_1_-H bonds homolysis (92.9–93.9 kcal/mol) from the point of thermodynamics. In addition, H^•^ (−102.3 kcal/mol) [28,29], Ru^IV^O^2+^ (−80.3 kcal/mol) [51], and PQ (−69.4 kcal/mol) [29] have the thermodynamic abilities to oxidize DH^−^ (64.4–65.7 kcal/mol), DH_2_ (breaking C_4_-H) (58.6–60.3 kcal/mol), DH^•^ (50.5–55.7 kcal/mol), DH_2_^•+^ (21.3–34.9 kcal/mol), and DH’^•^ (17.1–20.5 kcal/mol) via hydrogen atoms transfer from thermodynamics, meaning that dipines may be oxidated by heme enzyme, cytochrome P450, coenzyme Q (Co Q), and H^•^ under suitable oxidoreductase, by hydrogen atoms oxidation in vivo.

#### 2.3.4. Proton-Donating Properties

**Figure 8:**
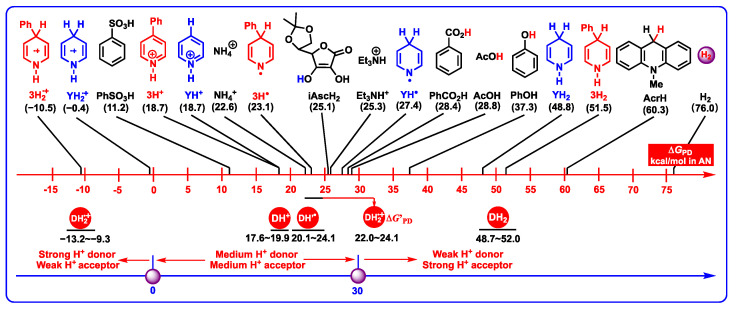
Thermodynamic proton-donating abilities of DH_2_, DH^+^, DH_2_^•+^, and DH’^•^, as well as proton-donating abilities of common acids in acetonitrile (kcal/mol).

In order to better understand the acidities of DH_2_ and related active intermediates, the thermodynamic proton-donating abilities of DH_2_, DH^+^, DH_2_^•+^, and DH’^•^, as well as the proton-donating abilities of common acids (PhSO_3_H, iAscH_2_, Et_3_NH^+^, PhCO_2_H, AcOH, AcrH, and H_2_) [43,44,45] in acetonitrile are presented in Figure 8.

Obviously, the proton-donating Gibbs free energy scale ranges from −13.2 to −9.3 kcal/mol for DH_2_^•+^ releasing protons from C_4_-H bonds, from 22.0 to 24.1 kcal/mol for DH_2_^•+^ releasing protons from N_1_-H bonds, from 17.6 to 19.9 kcal/mol for DH^+^, from 20.1 to 24.1 kcal/mol for DH’^•^, and from 48.7 to 52.0 kcal/mol for DH_2_, which discloses that the thermodynamic proton-donating abilities increase according to the order of DH_2_ (N_1_-H, 48.7–52.0 kcal/mol) < DH_2_^•+^ (N_1_-H, 22.0–24.1 kcal/mol) ≈ DH’^•^ (20.1–24.1 kcal/mol) < DH^+^ (17.6–19.9 kcal/mol) < DH_2_^•+^ (C_4_-H, −13.2–−9.3 kcal/mol). Based on their thermodynamic ranges, it could be suggested that DH_2_ (N_1_-H, 48.7–52.0 kcal/mol) belong to weak proton donors, DH_2_^•+^ (N_1_-H, 22.0–24.1 kcal/mol), DH’^•^ (20.1–24.1 kcal/mol), and DH^+^ (17.6–19.9 kcal/mol) belong to medium–strong proton donors, and DH_2_^•+^ (C_4_-H, −13.2–−9.3 kcal/mol) belong to strong proton donors.

The above thermodynamic analyses result in the following conclusions.

(1) DH_2_^•+^ (C_4_-H, −13.2–−9.3 kcal/mol) are the strongest organic acids among them. After the single-electron oxidation of DH_2_ (0.712–0.781 V vs. Fc), DH_2_^•+^ are extremely unstable intermediates that spontaneously release protons from C_4_-H bonds with significant thermodynamic potentials (−13.2–−9.3 kcal/mol).
(2) DH_2_ (48.7–52.0 kcal/mol) are the weakest organic acids in acetonitrile among them, indicating that the N_1_-H bond in DH_2_ is the weak polar bond. Since the proton affinity of hydride ions (H^−^) is determined as −76.0 kcal/mol in acetonitrile [41,42], the proton-abstracting reaction from N_1_-H bonds in DH_2_ molecules to H^−^ (DH_2_ + H^−^ → DH^−^ + H_2_) is thermodynamically favorable and extremely exothermic with −27.3 ≤ Δ*G*_PT_(DH_2_/H^−^) ≤ 24.0 kcal/mol.
(3) From Figure 8, the thermodynamic proton-abstracting abilities of common organic bases [43,44,45], consisting of iAscH^−^ (−25.1 kcal/mol), Et_3_N (−25.3 kcal/mol), NH_3_ (−22.6 kcal/mol), PhCO_2_^−^ (−28.4 kcal/mol), AcO^−^ (−28.8 kcal/mol), and PhO^−^ (−37.3 kcal/mol), are generally smaller than −40 kcal/mol. Although the proton abstraction reactions from DH_2_ to common organic bases are thermodynamically unfeasible (Δ*G*_PT_ > 0), the resulting DH^−^ (31.8–37.9 kcal/mol) are very excellent hydride donors, and the hydride transfers process from DH^−^ to hydride acceptors is extremely exothermic with Δ*G*_H_^−^_T_(DH^−^/H^−^-acceptor) << 0 kcal/mol. Therefore, it is reasonable to suppose that the concerted or successive proton and hydride (H^+^ + H^−^) transfer mechanism is thermodynamically feasible for the oxidation of dipines by hydride-acceptor/base pair in vivo.
(4) The common bases, such Et_3_N (−25.3 kcal/mol), NH_3_ (−22.6 kcal/mol) or amino acids, iAscH^−^ (−25.1 kcal/mol), PhCO_2_^−^ (−28.4 kcal/mol), AcO^−^ (−28.8 kcal/mol), and PhO^−^ (−37.3 kcal/mol) in vivo could easily absorb protons from the related intermediates of DH_2_, i.e., DH_2_^•+^ (C_4_-H, −13.2–−9.3 kcal/mol), DH_2_^•+^ (N_1_-H, 22.0–24.1 kcal/mol), DH’^•^ (20.1–24.1 kcal/mol), and DH^+^ (17.6–19.9 kcal/mol).
(5) According to structural features, DH_2_^•+^ (N_1_-H, 22.0–24.1 kcal/mol) are typical aromatic radical cations, while DH’^•^ (C_4_-H, 20.1–24.1 kcal/mol) are N-radical structures. However, they have thermodynamically similar proton-donating abilities for DH_2_^•+^ releasing protons from N_1_H bonds and DH’^•^ releasing protons from C_4_-H bonds. Interestingly, DH_2_^•+^ (C_4_-H, −13.2–−9.3 kcal/mol) are much stronger C-acids than DH’^•^ (C_4_-H, 20.1–24.1 kcal/mol) in acetonitrile.

#### 2.3.5. Two Hydrogen Ions (Atoms) Donating Properties

Examining the structural characteristics of NADH models, they are generally N-alkyl-1,4-dihydropyridines and act as hydride carriers [27,55]. Unlike common NADH models, the biggest difference between common NADH models (such as BNAH and AcrH) and DH_2_ or the Hantzsch ester (YH_2_) is that DH_2_ and YH_2_ have two hydrogen atoms at the N_1_-position and C_4_-position. During the aromatization process, DH_2_ and YH_2_ could release two hydrogen ions (atoms) or hydrogen gas from both N_1_-H and C_4_-H bonds. Therefore, the Hantzsch ester (YH_2_) has been widely used as an excellent hydrogenation reagent to reduce unsaturated compounds by offering two hydrogen ions (atoms) [56,57,58]. Furthermore, many studies also focused on the hydrogen storage properties of YH_2_ and related N-heterocycles [42]. In addition, some oxidoreductases, for example flavin coenzyme, coenzyme Q (Co Q), heme or nonheme iron coenzyme, and pyrroloquinoline quinone (PQQ) are also the two hydrogen ions (atoms) carriers in vivo. As a result, the thermodynamic abilities of DH_2_ releasing two hydrogen ions (atoms) or H_2_, Δ*G*_H_^−^_P_(DH_2_), Δ*G*_2H_(DH_2_), and Δ*G*_H2_(DH_2_), are vital thermodynamic parameters to evaluate the comprehensive reduction properties, and judge the oxidation feasibility of DH_2_ by two hydrogen ions (atoms) carrier enzymes in vivo.

**Figure 9:**
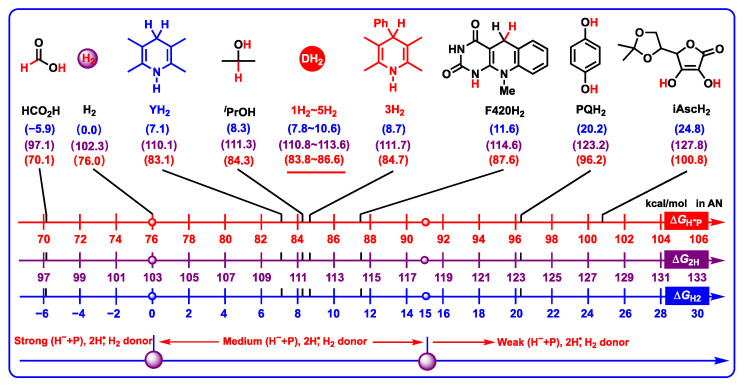
Thermodynamic abilities of DH_2_ and common hydrogen carriers releasing two hydrogen ions (H^−^ + H^+^) in the red brackets, releasing two hydrogen atoms (2H^•^) in the purple brackets, and releasing H_2_ in the blue brackets in acetonitrile (kcal/mol).

The thermodynamic abilities of DH_2_ and common hydrogen carriers (HCO_2_H, H_2_, YH_2_, F420H_2_, PQH_2_, and iAscH_2_) releasing two hydrogen ions (H^−^ + H^+^), two hydrogen atoms (2H^•^), or H_2_ in acetonitrile are shown in Figure 9 [27,43]. It is found from Figure 9 that the Gibbs free energies of DH_2_ releasing two hydrogen ions range from 83.8 to 86.6 kcal/mol, which belong to medium–strong two hydrogen ion donors.

According to the thermodynamic data, several valuable conclusions could be drawn. (1) The Δ*G*_H_^−^_P_(DH_2_) values (83.8–86.6 kcal/mol) are greater than those of H_2_ (76.0 kcal/mol) [41,42], meaning that DH_2_ are thermodynamically worse two hydrogen ions (atoms) reductants than H_2_, and H_2_ could hydrogenate D to regenerate DH_2_ in solution under suitable catalysts. (2) The Δ*G*_H_^−^_P_(DH_2_) values (83.8–86.6 kcal/mol) are slightly greater than Δ*G*_H_^−^_P_(YH_2_) (83.1 kcal/mol), illustrating that DH_2_ are thermodynamic alternatives of YH_2_ as two hydrogen ions (atoms) reductants in chemical reactions. (3) Since *p*-hydroquinone (PQH_2_) is a close model of Co Q, if the Δ*G*_H_^−^_P_(DH_2_) values (83.8–86.6 kcal/mol) are compared with the Δ*G*_H_^−^_P_(PQH_2_) value (96.2 kcal/mol) [29], it is discovered that DH_2_ are thermodynamically better two hydrogen ion donors than PQH_2_, and DH_2_ may be oxidated by Co Q via two hydrogen ions (atoms) transfer in vivo. (4) Due to the fact that Δ*G*_H_^−^_P_(DH_2_) values (83.8–86.6 kcal/mol) are much more negative than Δ*G*_H_^−^_P_(iAscH_2_) (100.8 kcal/mol) [27], DH_2_ are thermodynamically worse two hydrogen ions (atoms) donors, and DH_2_ may be oxidated by the oxidation state of ascorbic acid (Asc) in vivo. (5) Because the Δ*G*_H2_(DH_2_) values (7.8–10.6 kcal/mol) are greater than Δ*G*_H2_(H_2_) (0.0 kcal/mol), H_2_ release from DH_2_ is thermodynamically unfavorable, and DH_2_ are not hydrogen storage chemicals unless extra energy is being provided, such as light, electron, heat, or gas pressure.

### 2.4. Application of Thermodynamic Data to Evaluate the Redox Properties of DH_2_

**Figure 10:**
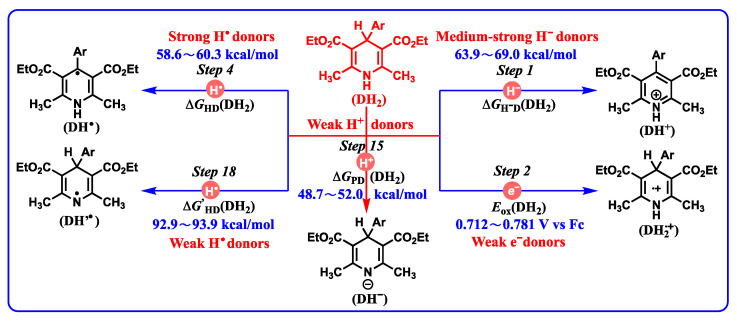
Thermodynamic abilities of five possible elementary steps for DH_2_ oxidation.

Without doubt, the thermodynamic cards of DH_2_ display the redox properties of DH_2_ to reveal the possible oxidative process of DH_2_ (Figure 3). For DH_2_, there are five possible initial oxidation pathways (Figure 10), including hydride oxidation (*Step 1*), single-electron oxidation (*Step 2*), hydrogen atom oxidation from the C_4_-H bond (*Step 4*), hydrogen atom oxidation from the N_1_-H bond (*Step 18*), and proton release from the N_1_-H bond (*Step 15*). As hydrogen atom donors or antioxidants, DH_2_ could release hydrogen atoms from the C_4_-H (*Step 4*) or N_1_-H (*Step 18*) bond. The Δ*G’*_HD_(DH_2_) values of *Step 18* (N_1_-H, 92.9–93.9 kcal/mol) are ~30.0 kcal/mol larger than the Δ*G’*_HD_(DH_2_) values of *Step 4* (C_4_-H, 58.6–60.3 kcal/mol). Thereby, it is reasonable to deduce that DH_2_ generally release hydrogen atoms from C_4_-H instead of N_1_-H during the antioxidant processes from thermodynamics.

Based on their thermodynamic results (*Steps 1–2*, *Step 4*, *Step 18*, and *Step 15*), DH_2_ are medium–strong hydride donors (*Step 1:* 63.9–69.0 kcal/mol), weak single electron donors (*Step 2:* 0.712–0.781 V vs. Fc), strong hydrogen atom donors for C_4_-H bond breaking (*Step 4:* 58.6–60.3 kcal/mol), weak hydrogen atom donors for N_1_-H bond breaking (*Step 18:* 92.9–93.9 kcal/mol), and weak proton donors (*Step 15:* 48.752.0 kcal/mol). It is supposed that (a) DH_2_ generally release hydrogen atoms from the C_4_-H bond instead of the N_1_-H bond in the radical oxidation process. (b) Generally, DH_2_ undergoing hydride or radical oxidations is thermodynamically favorable. (c) Since DH_2_ are thermodynamically very weak proton donors, the concerted or successive proton and hydride (H^+^ + H^−^) transfer mechanism is thermodynamically practicable. (d) The single-electron oxidation of DH_2_ is thermodynamically disadvantageous, and a high single-electron oxidant is needed.

### 2.5. Application of Thermodynamic Data of Important Intermediates to Possible Oxidative Mechanism Judgement

**Figure 11:**
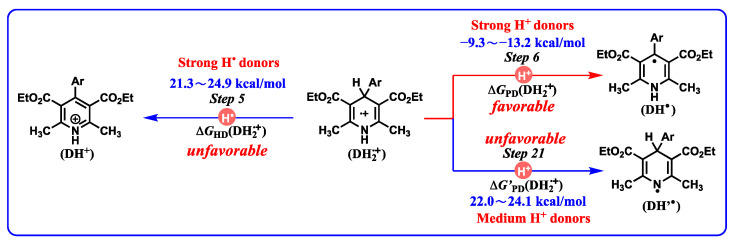
Thermodynamic analysis of possible oxidative process for intermediate DH_2_^•+^.

The thermodynamic cards of DH_2_ also could reveal the redox properties of key intermediates (Figure 3). For example, if the oxidation of DH_2_ is initiated by single oxidation, the thermodynamic data of resulting DH_2_^•+^ are noteworthy physical parameters to diagnose possible oxidation pathways. As organic acids, DH_2_^•+^ have two different pathways to donate protons (Figure 11), consisting of releasing protons from C_4_-H bonds (*Step 6*) and N_1_-H bonds (*Step 21*). When the thermodynamic potentials of *Step 6* and *Step 21* are compared, it is easy to find that the Δ*G*_PD_(DH_2_^•+^) values (−9.3–−13.2 kcal/mol) are 3and −35 kcal/mol more negative than Δ*G’*_PD_(DH_2_^•+^) (22.0–24.1 kcal/mol), indicating that, as proton donors, DH_2_^•+^ thermodynamically prefer to release protons from C_4_-H bonds instead of N_1_-H bonds, and the proton release from C_4_-H bonds in DH_2_^•+^ occurs spontaneously.

For DH_2_^•+^, there are three possible pathways to release hydrogen ions or atoms in all (Figure 11), including proton release from C_4_-H bonds (*Step 6*), proton release from N_1_-H bonds (*Step 21*), and hydrogen atom release from C_4_-H bonds (*Step 5*). According to their thermodynamic results (*Step 6*, *Step 21*, and *Step 5*), DH_2_^•+^ belong to strong proton donors for C_4_-H bond breaking (*Step 6:* −9.3–−13.2 kcal/mol), medium–strong proton donors for N_1_-H bond breaking (*Step 21:* 22.0–24.1 kcal/mol), and strong hydrogen atom donors for C_4_-H bond breaking (*Step 5:* 21.3–24.9 kcal/mol).

DH_2_^•+^ may act as proton donors or hydrogen atom donors, which depends on the properties of substrates. If the substrates are radicals, DH_2_^•+^ act as hydrogen atom donors. If the substrates are bases, DH_2_^•+^ act as proton donors. Most importantly, with a combination of the oxidation potentials of DH_2_ and the properties of resulting DH_2_^•+^ releasing hydrogen ions (atoms), the initial slight energy barrier (10–20 kcal/mol) of electron oxidation could be overcome by the subsequent protons transfer or hydrogen atoms transfer owning extreme thermodynamic driving forces (Δ*G* << 0).

### 2.6. Application of Thermodynamic Data of DH_2_ to Oxidative Mechanism Diagnosis between DH_2_ and iAsc

Since the thermodynamic cards of dipine models’ (DH_2_) aromatization on 21 potential elementary steps have been established, they provide a precious opportunity to investigate the possible oxidative mechanism of DH_2_ in vivo. Ascorbic acid (AscH_2_) is known as an excellent electron and hydrogen atom donor [52]; however, the oxidated ascorbic acid (Asc) is actually a potential two hydrogen ions (atoms) oxidant. Because of the ortho dicarbonyl feature, Asc has the property of *o*-benzoquinone [59], which is confirmed by the fact that Δ*G*_H_^−^_P_(iAscH_2_) (100.8 kcal/mol) [27] is slightly greater than Δ*G*_H_^−^_P_(PQH_2_) (96.2 kcal/mol) [29].

**Figure 12:**
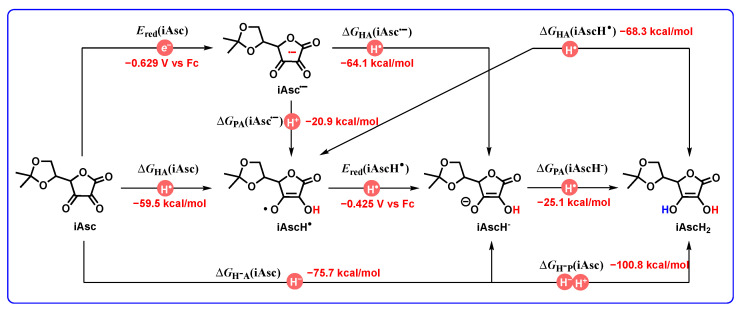
Thermodynamic card of iAsc accepting two hydrogen ions (atoms) on nine potential elementary steps.

**Figure 13:**
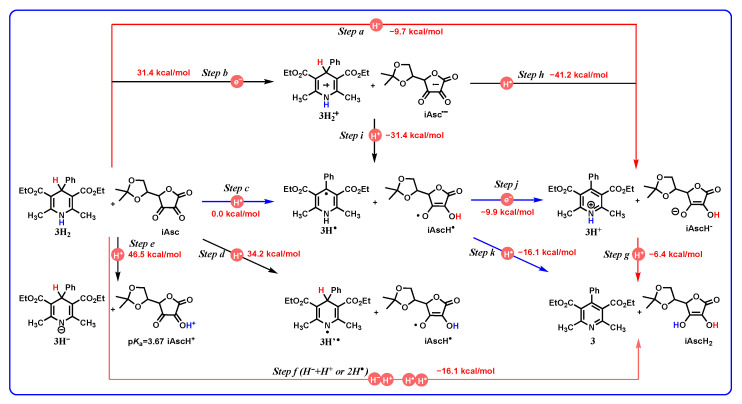
Thermodynamic analysis platform of elementary steps for the redox process between 3H_2_ and iAsc without any catalyst in acetonitrile.

Herein, the oxidative mechanism between 3H_2_ and iAsc is taken as an example to represent the application of the thermodynamic cards of DH_2_ to oxidative mechanism diagnosis. First of all, the thermodynamic card of iAsc accepting two hydrogen ions (atoms) on nine potential elementary steps is constructed based on our previous work (Figure 12). Subsequently, according to the thermodynamic cards of 3H_2_ and iAsc, a thermodynamic analysis platform of elementary steps for the redox process between 3H_2_ and iAsc without any catalyst in acetonitrile is established and shown in Figure 13.

From Figure 13, *Step 13* is the concerted two hydrogen ions (atoms) transfer step from 3H_2_ to iAsc, 3H_2_ + iAsc → 3 + iAscH_2_, and the overall Gibbs free energy of two hydride ions (atoms) transfer from 3H_2_ to iAsc for *Step 13* is −16.1 kcal/mol, which means the oxidation of 3H_2_ by iAsc is thermodynamically feasible. Moreover, as can be seen from Figure 13, six possible elementary steps are involved in the initial oxidation of 3H_2_ by iAsc. *Step a* is the hydride transfer step from 3H_2_ to iAsc, 3H_2_ + iAsc → 3H^+^ + iAscH^−^, and the Gibbs free energy of *Step a* is −9.7 kcal/mol. *Step b* is the electron transfer step from 3H_2_ to iAsc, 3H_2_ + iAsc → 3H^•+^ + iAsc^•−^, and the Gibbs free energy of *Step b* is 31.4 kcal/mol. *Steps c* and *d* are the hydrogen atom transfer step from C_4_-H and N_1_-H bonds in 3H_2_ to iAsc, respectively, 3H_2_ + iAsc → 3H^•^ + iAscH^•^ and 3H_2_ + iAsc → 3H’^•^ + iAscH^•^, and the Gibbs free energy of *Steps c* and *d* are 0.0 and 34.2 kcal/mol. *Step e* is the proton transfer step from 3H_2_ to iAsc, 3H_2_ + iAsc → 3H^−^ + iAscH^+^, and the Gibbs free energy of *Step e* is 46.5 kcal/mol. Finally, *Step f* is the concerted two hydrogen ions (atoms) transfer step from 3H_2_ to iAsc, and the Gibbs free energy of *Step f* is −16.1 kcal/mol.

According to the thermodynamic potentials of *Steps a–f*, the Gibbs free energies of *Step b* (31.4 kcal/mol), *Step d* (34.2 kcal/mol), and *Step e* (46.8 kcal/mol) are greater than 30 kcal/mol, which implies that the oxidation of 3H_2_ could not occur through electron transfer (*Step b*), hydrogen transfer from the N_1_-H bond (*Step d*), and proton transfer (*Step e*). Since the Gibbs free energies of *Step a* (−9.7 kcal/mol), *Step c* (0.0 kcal/mol), and *Step f* (−16.1 kcal/mol) are less than or equal to zero, the oxidation of 3H_2_ may experience an initial hydride (*Step a*), hydrogen atom from C_4_-H (*Step c*), and concerted two hydrogen ions (atoms) transfer step (*Step f*).

Further investigating the thermodynamics of possible hydrogen atom oxidation processes, there are two different pathways for the oxidation of 3H_2_ by iAsc overall. The first is the H^•^ + e + H^+^ pathway (*Step c–Step j–Step g*), and the second is the H^•^ + H^•^ pathway (*Step c–Step k*). Due to the fact that all of the Gibbs free energies of the above five elementary steps are less than or equal to zero (−16.1–0.0 kcal/mol), it can be assumed that these two hydrogen-atom-initiated pathways, *Step c–Step j–Step g* and *Step c–Step k*, are thermodynamically feasible.

Additionally, the Gibbs free energies of direct hydride transfer from 3H_2_ to iAsc and proton transfer from 3H^+^ to iAscH^−^, *Step a* (hydride transfer, −9.7 kcal/mol) and *Step g* (proton transfer, −6.4 kcal/mol), are much less than zero, and it can be inferred that the oxidation process of 3H_2_ and iAsc undergoing successive hydride (*Step a,* −9.7 kcal/mol) and proton transfer (*Step g*, −6.4 kcal/mol) is thermodynamically feasible.

Similarly, the oxidation of 3H_2_ by iAsc processing concerted two hydrogen ions (atoms) transfer (*Step f*, −16.1 kcal/mol) is also thermodynamically feasible. Although the concerted two hydrogen ions (atoms) transfer (*Step f*) is thermodynamically feasible, 3H_2_ and iAsc molecules do not completely match each other in space structure and binding site during transition state. Therefore, the concerted two hydrogen ions (atoms) transfer (*Step f*) process is reasonably ruled out. It may be that in complex environments within the body, the hydride-acceptor/amino-acid residue pairs, two radicals, or benzoquinones could oxidize dipines through a concerted two hydrogen ions (atoms) transfer step.

In summary, the oxidation of 3H_2_ by iAsc may experience three possible pathways, that is, H^−^ + H^+^ (*Step a–step g*), H^•^ + *e* + H^+^ (*Step c–Step j–Step g*), and H^•^ + H^•^ (*Step c–Step k*). But which one pathway or pathways are the real oxidation mechanism needs further experimental verification in the lab. Beyond a doubt, the thermodynamic analyses of the redox process between 3H_2_ and iAsc provide us with a unique perspective into the dipines’ aromatization by quinones in vivo.

## 3. Materials and Methods

Prediction Methods. The p*K*_a_ values of DH^+^ and YH^+^ in acetonitrile were predicted by the method developed by Luo and coworkers in 2020 at http://pka.luoszgroup.com/prediction (accessed on 9 January 2024). Prediction methods: XGBoost with RMSE = 1.79 and r^2^ = 0.918 (80:20 train test split).

## 4. Conclusions

For dipines, their core skeleton is the 1,4-dihydropyridine structure, which is a key structural factor for biological activity. During the metabolism of dipines in vivo, 1,4-dihydropyridine rings are oxidized to release formal two hydrogen ions (atoms) and generate the aromatic pyridines, and six possible intermediates and 21 potential elementary steps are involved in the process. In this work, 4-substituted-phenyl-2,6-dimethyl-3,5-diethyl-formate-1,4-dihydropyridines (DH_2_) are refined as the structurally closest dipine models, and the thermodynamic cards of these dipine models’ aromatization on 21 potential elementary steps in acetonitrile have been established.

Based on the thermodynamic cards, the thermodynamic properties of dipine models and related intermediates acting as electrons, hydrides, hydrogen atoms, protons, and two hydrogen ions (atoms) donors are discussed. Several valuable conclusions are made as follows.

(1) Electron-donating properties. The electron-donating abilities increase in the order of DH_2_ < DH^−^ < DH^•^ < D^•−^. DH_2_, DH^−^, DH^•^, and D^•−^ are recognized as the weak electron donors, medium–strong electron donors, strong electron donors, and very strong electron donors, respectively.
(2) Hydride-donating properties. DH_2_ and DH^−^ belong to thermodynamically medium–strong and strong hydride donors, respectively. It can be inferred that dipines may be oxidated by heme enzyme, cytochrome P450, flavin coenzyme, coenzyme Q, and H^+^ under suitable oxidoreductase by hydride oxidation in vivo.
(3) Hydrogen-atom-donating properties. The thermodynamic hydrogen-atom-donating abilities increase in the order of DH_2_ (N_1_-H) < DH^−^ < DH_2_ (C_4_-H) < DH^•^< DH_2_^•+^ < DH’^•^. Dipines may be oxidated by heme enzyme, cytochrome P450, coenzyme Q (Co Q), and H^•^ under suitable oxidoreductase, by hydrogen atom oxidation in vivo.
(4) Proton-donating properties. DH_2_ belong to weak proton donors, DH_2_^•+^ (N_1_-H), DH’^•^, and DH^+^ belong to medium–strong proton donors, and DH_2_^•+^ (C_4_-H) belong to strong proton donors. After DH_2_ are oxidized by hydride acceptors, the acidities of resulting DH^+^ should be considered when the organic bases are involved in the reaction system.
(5) Two hydrogen ions (atoms) donating properties. DH_2_ belong to medium–strong two hydrogen ion donors. H_2_ could hydrogenate D to regenerate DH_2_ in solution under suitable catalysts. DH_2_ maybe oxidated by Co Q via two hydrogen ions (atoms) transfer in vivo.

Moreover, the thermodynamic cards were applied to evaluate the redox properties and judge the possible oxidative mechanism of dipine models. The important conclusions are drawn as follows.

(1) Application in evaluating the redox properties of DH_2_: DH_2_ generally release hydrogen atoms from the C_4_-H bond instead of the N_1_-H bond in the radical oxidation process. Generally, DH_2_ undergoing hydride or radical oxidations is thermodynamically favorable. Since DH_2_ are thermodynamically very weak proton donors, the concerted or successive proton and hydride (H^+^ + H^−^) transfer mechanism is thermodynamically practicable. The single-electron oxidation of DH_2_ is thermodynamically disadvantageous, and a high single-electron oxidant is needed.
(2) Application in judging the possible oxidative mechanism for important intermediates: DH_2_^•+^ may act as proton donors or hydrogen atom donors, which depends on the properties of substrates. If the substrates are radicals, DH_2_^•+^, act as hydrogen atom donors. If the substrates are bases, DH_2_^•+^, act as proton donors. Most importantly, with a combination of the oxidation potentials of DH_2_ and the properties of resulting DH_2_^•+^ releasing hydrogen ions (atoms), the initial slight energy barrier (10–20 kcal/mol) of electron oxidation could be overcome by the subsequent protons transfer or hydrogen atoms transfer owning extreme thermodynamic driving forces (Δ*G* << 0).
(3) The application of the thermodynamic data of DH_2_ to oxidative mechanism diagnosis between DH_2_ and iAsc: Because of the ortho dicarbonyl feature, Asc has the property of *o*-benzoquinone. Therefore, the oxidative mechanism between 3H_2_ and iAsc is taken as an example to represent the application of the thermodynamic cards of DH_2_ to oxidative mechanism diagnosis. Based on thermodynamic analyses, the oxidation of 3H_2_ by iAsc may experience three possible pathways, that is, H^−^ + H^+^, H^•^ + *e* + H^+^, and H^•^ + H^•^.

The thermodynamic data of dipine models’ aromatization suggests that the oxidative metabolism of dipine drugs is unexpectedly complex, and may involve many active intermediates and potential elementary steps under the conditions of various oxidoreductases in vivo. Therefore, the effects and levels of electrons and hydrogen atoms, as well as hydride oxidoreductases may need sustained attention or detection during the treatment period. Without a doubt, the thermodynamic cards of dipine models could help us to understand the redox properties of DH_2_ and related intermediates, and diagnose the possible mechanism and pathways of dipine metabolism in vivo.

## Figures and Tables

**Table 2:** Thermodynamic results of dipine models (DH_2_) and Hantzsch ester (YH_2_) aromatization on 21 elementary steps.

*Step X*	Parameters	1H_2_	2H_2_	3H_2_	4H_2_	5H_2_	YH_2_
*Step 1*	Δ*G*_H_^−^_D_(DH_2_)	63.9	64.7	66.0	66.9	69.0	64.4
*Step 2*	*E*_ox_(DH_2_)	0.712	0.721	0.731	0.745	0.781	0.479
*Step 3*	*E*_ox_(DH^•^)	−0.980	−0.932	−0.856	−0.775	−0.655	−1.112
*Step 4*	Δ*G*_HD_(DH_2_)	60.3	60.0	59.5	58.6	57.9	63.8
*Step 5*	Δ*G*_HD_(DH_2_^•+^)	21.3	21.9	22.9	23.5	24.9	27.1
*Step 6*	Δ*G*_PD_(DH_2_^•+^)	−9.3	−9.9	−10.5	−11.8	−13.2	−0.4
*Step 7*	Δ*G*_H_^−^_D_(DH^−^)	31.8	32.2	33.2	34.6	37.9	34.3
*Step 8*	*E*_ox_(DH^−^)	−0.497	−0.491	−0.479	−0.444	−0.382	−0.695
*Step 9*	*E*_ox_(D^•−^)	−2.611	−2.580	−2.528	−2.453	−2.289	−2.446
*Step 10*	Δ*G*_HD_(DH^−^)	65.7	65.4	65.2	64.9	64.4	64.4
*Step 11*	Δ*G*_HD_(DH’^•^)	17.1	17.3	18.0	18.6	20.5	24.1
*Step 12*	Δ*G*_PD_(DH’^•^)	24.1	23.6	23.1	22.0	20.1	27.4
*Step 13*	Δ*G*_PD_(DH^+^)	19.9	19.5	18.7	17.6	17.6	18.7
*Step 14*	Δ*G*_H_^−^_P_(DH_2_)	83.8	84.2	84.7	84.5	86.6	83.1
*Step 15*	Δ*G*_PD_(DH_2_)	52.0	52.0	51.5	49.9	48.7	48.8
*Step 16*	Δ*G*_2H_(DH_2_)	110.8	111.2	111.7	111.5	113.6	110.1
*Step 17*	Δ*G*_HD_(DH^•^)	50.5	51.2	52.2	52.9	55.7	46.3
*Step 18*	Δ*G’*_HD_(DH_2_)	93.7	93.9	93.7	92.9	93.1	86.0
*Step 19*	Δ*G*_H2_(DH_2_)	7.8	8.2	8.7	8.5	10.6	7.1
*Step 20*	Δ*G*_HT_(DH^•^)	33.4	33.9	34.2	34.3	35.2	22.2
*Step 21*	Δ*G’*_PD_(DH_2_^•+^)	24.1	24.0	23.7	22.5	22.0	21.8

Note: The unit of potentials is V vs. Fc, and the unit of Gibbs free energies is kcal/mol. *E*_ox_(DH_2_), *E*_ox_(DH^•^), *E*_ox_(DH^−^), and *E*_ox_(D^•−^) values are taken from [38].

## Data Availability

The data underlying this study are available in the published article.

## Supplementary Materials

The following supporting information can be downloaded at: https://www.mdpi.com/article/10.3390/molecules29153706/s1. The prediction method of p*K*_a_, original data of p*K*_a_ prediction, and thermodynamic cards of DH_2_ and YH_2_ are available in Supporting Information. Reference [40] are cited in the supplementary materials.
