# Supplementary material for: Establishing the Thermodynamic Cards of Dipine Models’ Oxidative Metabolism on 21 Potential Elementary Steps
Source: Molecules. 2024 Aug 5;29(15):3706. doi: 10.3390/molecules29153706 (PMC11313972; doi:10.3390/molecules29153706)
Supplement: Supplementary file 1 [file molecules-29-03706-s001.zip › molecules-3108006-supplementary.pdf]

The Supporting Information For

Establishing the Thermodynamic Cards of Dipine Models' Oxidative Metabolism on 21 Potential Elementary Steps

Guang-Bin Shen [1] Shun-Hang Gao [1], Yan-Wei Jia [1], Xiao-Qing Zhu [2,*] and Bao-Chen Qian [1,*]

[1]*College of Medical Engineering, Jining Medical University, Jining, Shandong, 272000, P. R. China.*

[2]*The State Key Laboratory of Elemento-Organic Chemistry, Department of Chemistry, Nankai University, Tianjin 300071, China*

* *Correspondence: xqzhu@nankai.edu.cn; qianbc@mail.jnmc.edu.cn;*

Contents

The prediction method of pK_a *S2*

The original data of pK_a predictions.. *S3*

Thermodynamic cards.. *S6*

The prediction method of pK_a for DH^+ and YH^+ in acetonitrile

The pK_a values of DH^+ and YH^+ in acetonitrile are predicted using the method developed by Luo and coworkers in 2020 at *http://pka.luoszgroup.com/prediction* (Yang, Q.; Li, Y.; Yang, J.-D.; Liu, Y.; Zhang, L.; Luo, S.; Cheng, J.-P. Holistic prediction of pK_a in diverse solvents based on machine learning approach. *Angew. Chem., Int. Ed.* **2020**, *59*, 19282-19291.). Prediction Methods: XGBoost with RMSE = 1.79 and r^2 = 0.918 (80:20 train test split). The prediction method was developed based on machine learning approach and well verified with an absolute error of 0.87 pK_a units based on 15,338 experimental pK_a data in Luo's work.

The original data of pK_a predictions

1. The predicted pK_a of $1H^+$ in different solvents ↓

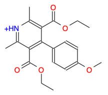

Identification

IUPAC Name:	null
CAS Registry Number(s):	null
Molecular Formula:	C20H24NO5+
Mol. Wt:	358.414
Exact Mass:	358.165
LogP:	3.14654
TPSA:	75.97
MolMR:	96.3727
Smiles:	CCOC(=O)c1c(C)[nH+]c(C)c(C(=O)OCC)c1-c1ccc(OC)cc1
InChIKey:	QHLHUPBFLVOBLD-UHFFFAOYSA-O
InChI:	1S/C20H23NO5/c1-6-25-19(22)16-12(3)21-13(4)17(20(23)26-7-2)18(16)14-8-10-15(24-5)11-9-14/h8-11H,6-7H2,1-5H3/p+1

solvent	XGBoost	Experimental Data
H2O	4.58	-
DMSO	11.41	-
EtOH_50%	5.86	-
AN	14.54	-
MeOH	8.66	-

Please Cite:
Qi Yang, Yao Li, Jin-Dong Yang, Yidi Liu, Long Zhang*, Sanzhong Luo*, Jin-Pei Cheng, Holistic Prediction of pK_a in Diverse Solvents Based on Machine Learning Approach. *Angew. Chem. Int. Ed.* **2020**, *59*, 19282-19291.

1. Abbreviations of solvents: H_2O: Water; DMSO: Dimethyl sulfoxide; EtOH_50%: Ethanol:Water=50:50; AN: Acetonitrile; MeOH: Methanol.

2. Prediction methods: XGBoost with RMSE=1.79 and r^2=0.918 (80:20 train test split).

3. Experimental data: experimental data comes from the sub-database of **iBonD**, for more details please click **http://ibond.nankai.edu.cn.**

4. Recommended pK_a ranges: H_2O: -2~16; DMSO: 5~35; EtOH_50%: 0~20; AN: 5~30; MeOH: 0~18.

5. Note: for special molecule's pK_a which is out of the solvent leveling range, the experimental value is unreliable, [illegible]

2. The predicted pK_a of $2H^+$ in different solvents ↓

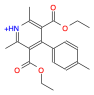

Identification

IUPAC Name:	null
CAS Registry Number(s):	null
Molecular Formula:	C20H24NO4+
Mol. Wt:	342.415
Exact Mass:	342.17
LogP:	3.44636
TPSA:	66.74
MolMR:	94.5577
Smiles:	CCOC(=O)c1c(C)[nH+]c(C)c(C(=O)OCC)c1-c1ccc(C)cc1
InChIKey:	AYVZSACBHVWDPY-UHFFFAOYSA-O
InChI:	1S/C20H23NO4/c1-6-24-19(22)16-13(4)21-14(5)17(20(23)25-7-2)18(16)15-10-8-12(3)9-11-15/h8-11H,6-7H2,1-5H3/p+1

solvent	XGBoost	Experimental Data
H2O	3.85	-
DMSO	10.63	-
EtOH_50%	4.99	-
AN	14.22	-
MeOH	7.63	-

Please Cite:
Qi Yang, Yao Li, Jin-Dong Yang, Yidi Liu, Long Zhang*, Sanzhong Luo*, Jin-Pei Cheng, Holistic Prediction of pK_a in Diverse Solvents Based on Machine Learning Approach. *Angew. Chem. Int. Ed.* **2020**, *59*, 19282-19291.

1. Abbreviations of solvents: H_2O: Water; DMSO: Dimethyl sulfoxide; EtOH_50%: Ethanol:Water=50:50; AN: Acetonitrile; MeOH: Methanol.

2. Prediction methods: XGBoost with RMSE=1.79 and r^2=0.918 (80:20 train test split).

3. Experimental data: experimental data comes from the sub-database of **iBonD**, for more details please click **http://ibond.nankai.edu.cn.**

4. Recommended pK_a ranges: H_2O: -2~16; DMSO: 5~35; EtOH_50%: 0~20; AN: 5~30; MeOH: 0~18.

5. Note: for special molecule's pK_a which is out of the solvent leveling range, the experimental value is unreliable, [illegible]

3. The predicted pK_a of $3H^+$ in different solvents ↓

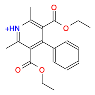

Identification

IUPAC Name:	null
CAS Registry Number(s):	null
Molecular Formula:	C19H22NO4+
Mol. Wt:	328.388
Exact Mass:	328.154
LogP:	3.13794
TPSA:	66.74
MolMR:	89.8207
Smiles:	CCOC(=O)c1c(C)[nH+]c(C)c(C(=O)OCC)c1-c1ccccc1
InChIKey:	VQDKSJMDSZZJER-UHFFFAOYSA-O
InChI:	1S/C19H21NO4/c1-5-23-18(21)15-12(3)20-13(4)16(19(22)24-6-2)17(15)14-10-8-7-9-11-14/h7-11H,5-6H2,1-4H3/p+1

solvent	XGBoost	Experimental Data
H2O	3.81	-
DMSO	10.72	-
EtOH_50%	4.98	-
AN	13.66	-
MeOH	7.76	-

Please Cite:
Qi Yang, Yao Li, Jin-Dong Yang, Yidi Liu, Long Zhang*, Sanzhong Luo*, Jin-Pei Cheng, Holistic Prediction of pK_a in Diverse Solvents Based on Machine Learning Approach. *Angew. Chem. Int. Ed.* **2020**, *59*, 19282-19291.

1. Abbreviations of solvents: H_2O: Water; DMSO: Dimethyl sulfoxide; EtOH_50%: Ethanol:Water=50:50; AN: Acetonitrile; MeOH: Methanol.

2. Prediction methods: XGBoost with RMSE=1.79 and r^2=0.918 (80:20 train test split).

3. Experimental data: experimental data comes from the sub-database of **iBonD**, for more details please click **http://ibond.nankai.edu.cn.**

4. Recommended pK_a ranges: H_2O: -2~16; DMSO: 5~35; EtOH_50%: 0~20; AN: 5~30; MeOH: 0~18.

5. Note: for special molecule's pK_a which is out of the solvent leveling range, the experimental value is unreliable,

4. The predicted pK_a of $4H^+$ in different solvents ↓

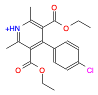

Identification

IUPAC Name:	null
CAS Registry Number(s):	null
Molecular Formula:	C19H21ClNO4+
Mol. Wt:	362.833
Exact Mass:	362.115
LogP:	3.79134
TPSA:	66.74
MolMR:	94.8307
Smiles:	CCOC(=O)c1c(C)[nH+]c(C)c(C(=O)OCC)c1-c1ccc(Cl)cc1
InChIKey:	RNKJOFQOYOOVHP-UHFFFAOYSA-O
InChI:	1S/C19H20ClNO4/c1-5-24-18(22)15-11(3)21-12(4)16(19(23)25-6-2)17(15)13-7-9-14(20)10-8-13/h7-10H,5-6H2,1-4H3/p+1

solvent	XGBoost	Experimental Data
H2O	3.70	-
DMSO	10.30	-
EtOH_50%	5.14	-
AN	12.88	-
MeOH	8.01	-

Please Cite:
Qi Yang, Yao Li, Jin-Dong Yang, Yidi Liu, Long Zhang*, Sanzhong Luo*, Jin-Pei Cheng, Holistic Prediction of pK_a in Diverse Solvents Based on Machine Learning Approach. *Angew. Chem. Int. Ed.* **2020**, *59*, 19282-19291.

1. Abbreviations of solvents: H_2O: Water; DMSO: Dimethyl sulfoxide; EtOH_50%: Ethanol:Water=50:50; AN: Acetonitrile; MeOH: Methanol.

2. Prediction methods: XGBoost with RMSE=1.79 and r^2=0.918 (80:20 train test split).

3. Experimental data: experimental data comes from the sub-database of **iBonD**, for more details please click **http://ibond.nankai.edu.cn.**

4. Recommended pK_a ranges: H_2O: -2~16; DMSO: 5~35; EtOH_50%: 0~20; AN: 5~30; MeOH: 0~18.

5. Note: for special molecule's pK_a which is out of the solvent leveling range, the experimental value is unreliable,

5. The predicted pK_a of $5H^+$ in different solvents ↓

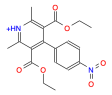

Identification

IUPAC Name:	null
CAS Registry Number(s):	null
Molecular Formula:	C19H21N2O6+
Mol. Wt:	373.385
Exact Mass:	373.139
LogP:	3.04614
TPSA:	109.88
MolMR:	96.4751
Smiles:	CCOC(=O)c1c(C)[nH+]c(C)c(C(=O)OCC)c1-c1ccc([N+](=O)[O-])cc1
InChIKey:	JNHHAORIYCGJKA-UHFFFAOYSA-O
InChI:	1S/C19H20N2O6/c1-5-26-18(22)15-11(3)20-12(4)16(19(23)27-6-2)17(15)13-7-9-14(10-8-13)21(24)25/h7-10H,5-6H2,1-4H3/p+1

solvent	XGBoost	Experimental Data
H2O	2.69	-
DMSO	8.20	-
EtOH_50%	4.20	-
AN	12.83	-
MeOH	7.23	-

Please Cite:
Qi Yang, Yao Li, Jin-Dong Yang, Yidi Liu, Long Zhang*, Sanzhong Luo*, Jin-Pei Cheng, Holistic Prediction of pK_a in Diverse Solvents Based on Machine Learning Approach. *Angew. Chem. Int. Ed.* **2020**, *59*, 19282-19291.

1. Abbreviations of solvents: H_2O: Water; DMSO: Dimethyl sulfoxide; EtOH_50%: Ethanol:Water=50:50; AN: Acetonitrile; MeOH: Methanol.

2. Prediction methods: XGBoost with RMSE=1.79 and r^2=0.918 (80:20 train test split).

3. Experimental data: experimental data comes from the sub-database of **iBonD**, for more details please click **http://ibond.nankai.edu.cn.**

4. Recommended pK_a ranges: H_2O: -2~16; DMSO: 5~35; EtOH_50%: 0~20; AN: 5~30; MeOH: 0~18.

5. Note: for special molecule's pK_a which is out of the solvent leveling range, the experimental value is unreliable, [illegible]

6. The predicted pK_a of HEH^+ in different solvents ↓

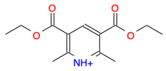

Identification

IUPAC Name:	null
CAS Registry Number(s):	null
Molecular Formula:	C13H18NO4+
Mol. Wt:	252.29
Exact Mass:	252.123
LogP:	1.47094
TPSA:	66.74
MolMR:	64.3847
Smiles:	CCOC(=O)c1cc(C(=O)OCC)c(C)[nH+]c1C
InChIKey:	DIIWSYPKAJVXBV-UHFFFAOYSA-O
InChI:	1S/C13H17NO4/c1-5-17-12(15)10-7-11(13(16)18-6-2)9(4)14-8(10)3/h7H,5-6H2,1-4H3/p+1

solvent	XGBoost	Experimental Data
H2O	2.99	2.96
DMSO	10.11	-
EtOH_50%	4.36	-
AN	13.65	-
MeOH	7.19	-

Please Cite:
Qi Yang, Yao Li, Jin-Dong Yang, Yidi Liu, Long Zhang*, Sanzhong Luo*, Jin-Pei Cheng, Holistic Prediction of pK_a in Diverse Solvents Based on Machine Learning Approach. *Angew. Chem. Int. Ed.* **2020**, *59*, 19282-19291.

1. Abbreviations of solvents: H_2O: Water; DMSO: Dimethyl sulfoxide; EtOH_50%: Ethanol:Water=50:50; AN: Acetonitrile; MeOH: Methanol.

2. Prediction methods: XGBoost with RMSE=1.79 and r^2=0.918 (80:20 train test split).

3. Experimental data: experimental data comes from the sub-database of **iBonD**, for more details please click **http://ibond.nankai.edu.cn.**

4. Recommended pK_a ranges: H_2O: -2~16; DMSO: 5~35; EtOH_50%: 0~20; AN: 5~30; MeOH: 0~18.

Thermodynamic cards of 1H$_2$~5H$_2$ and YH$_2$ in acetonitrile

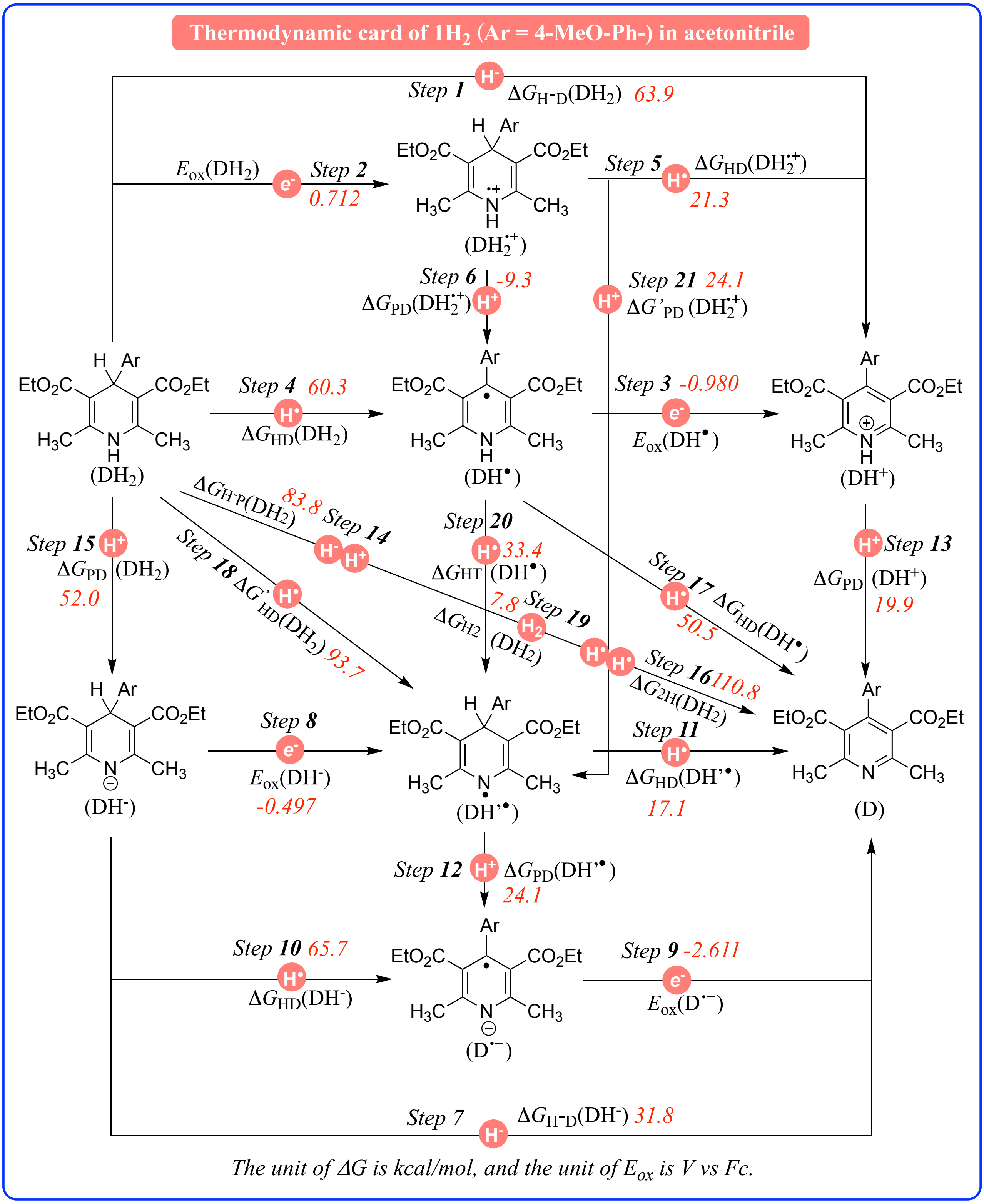

Figure S1. *Thermodynamic card of 1H$_2$ in acetonitrile*

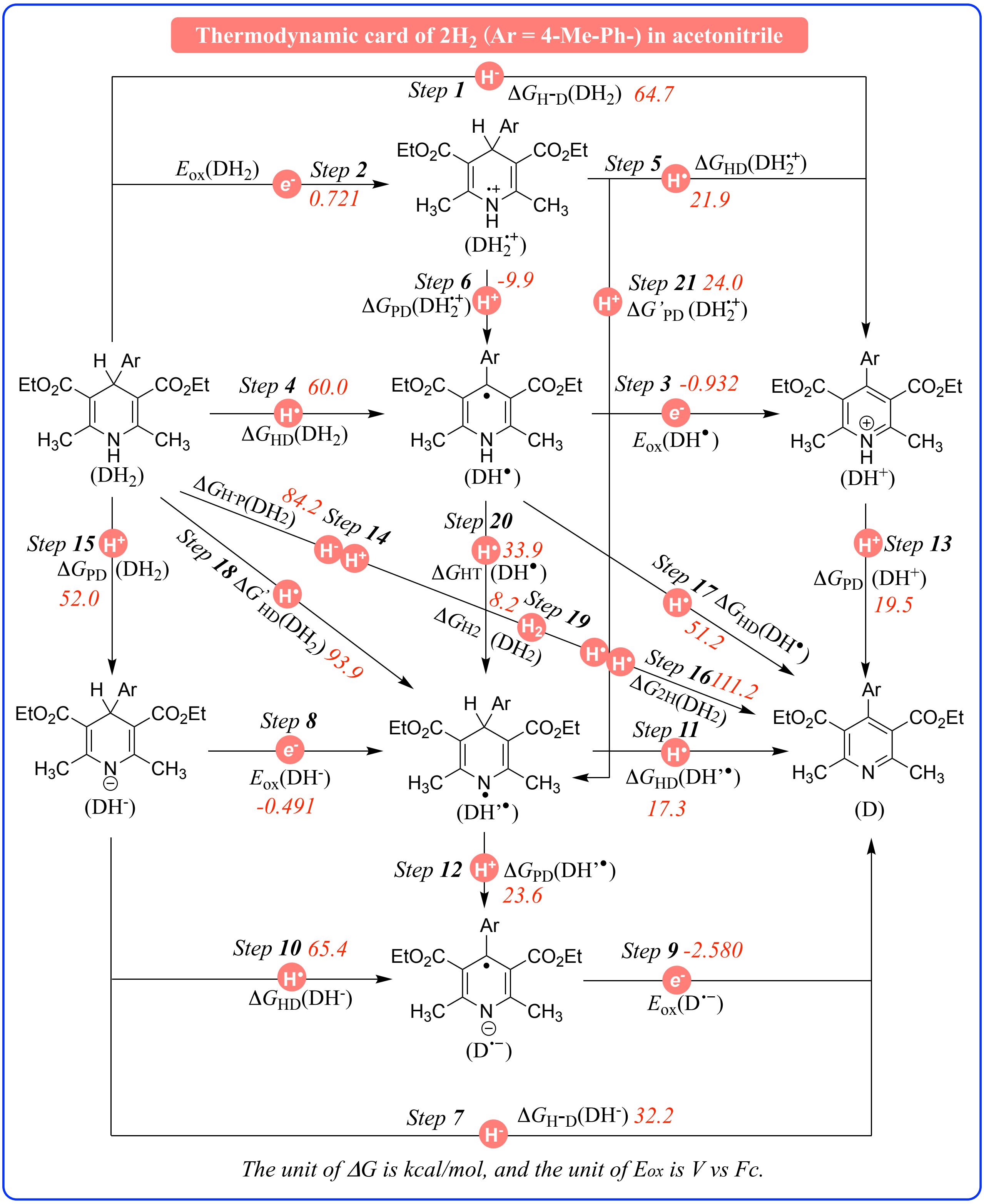

Figure S2. *Thermodynamic card of $2H_2$ in acetonitrile*

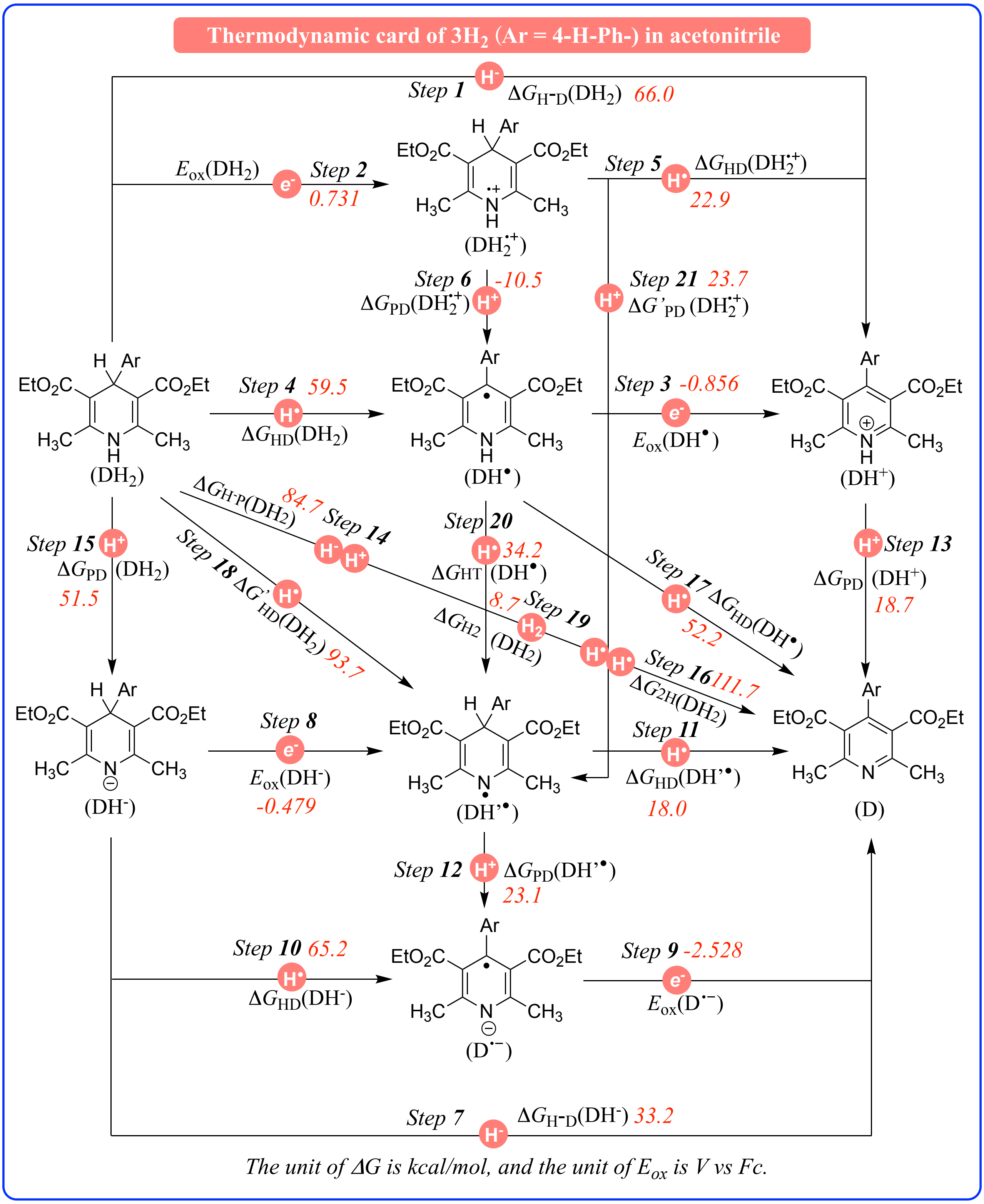

Figure S3. *Thermodynamic card of 3H₂ in acetonitrile*

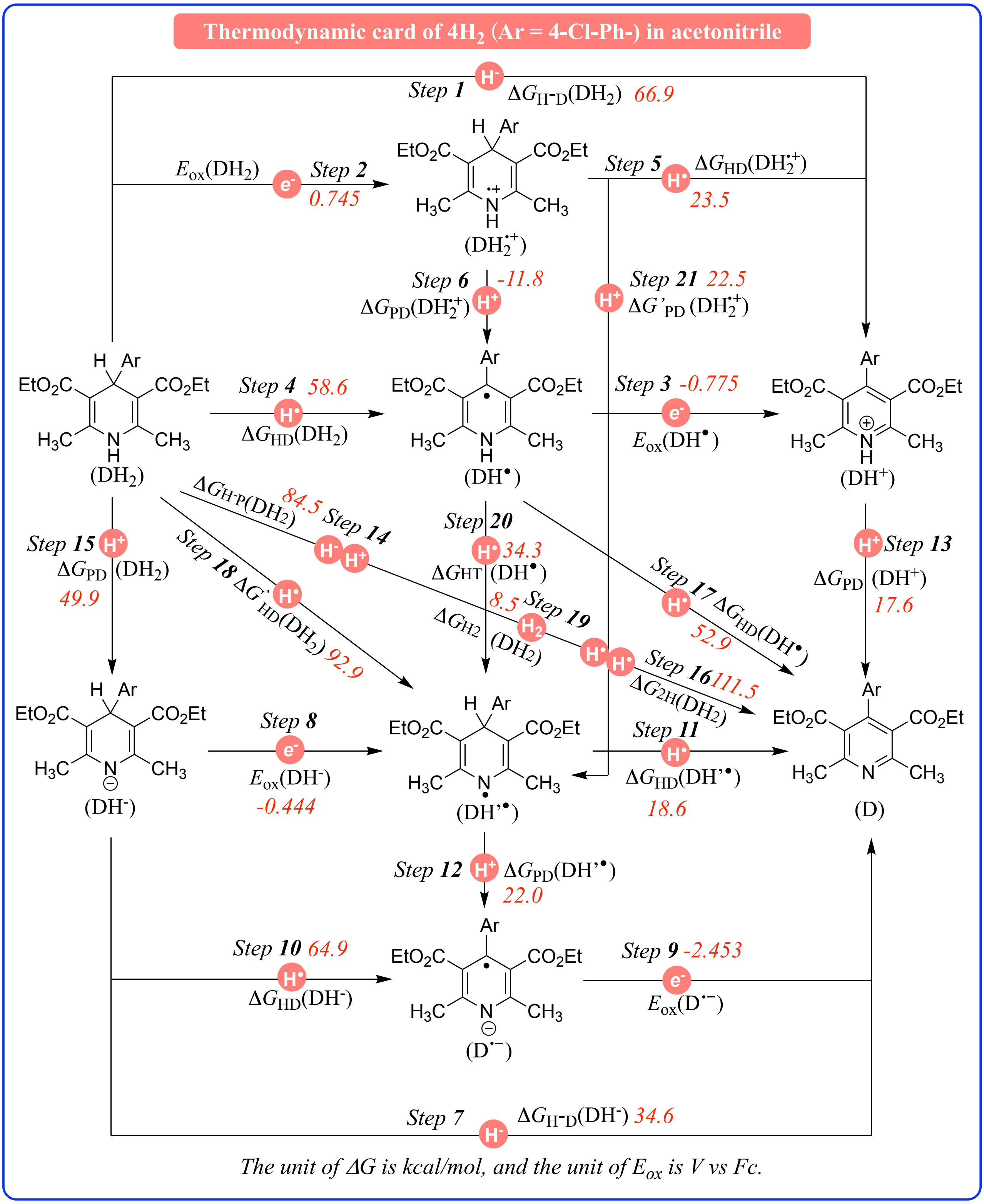

Figure S4. *Thermodynamic card of $4H_2$ in acetonitrile*

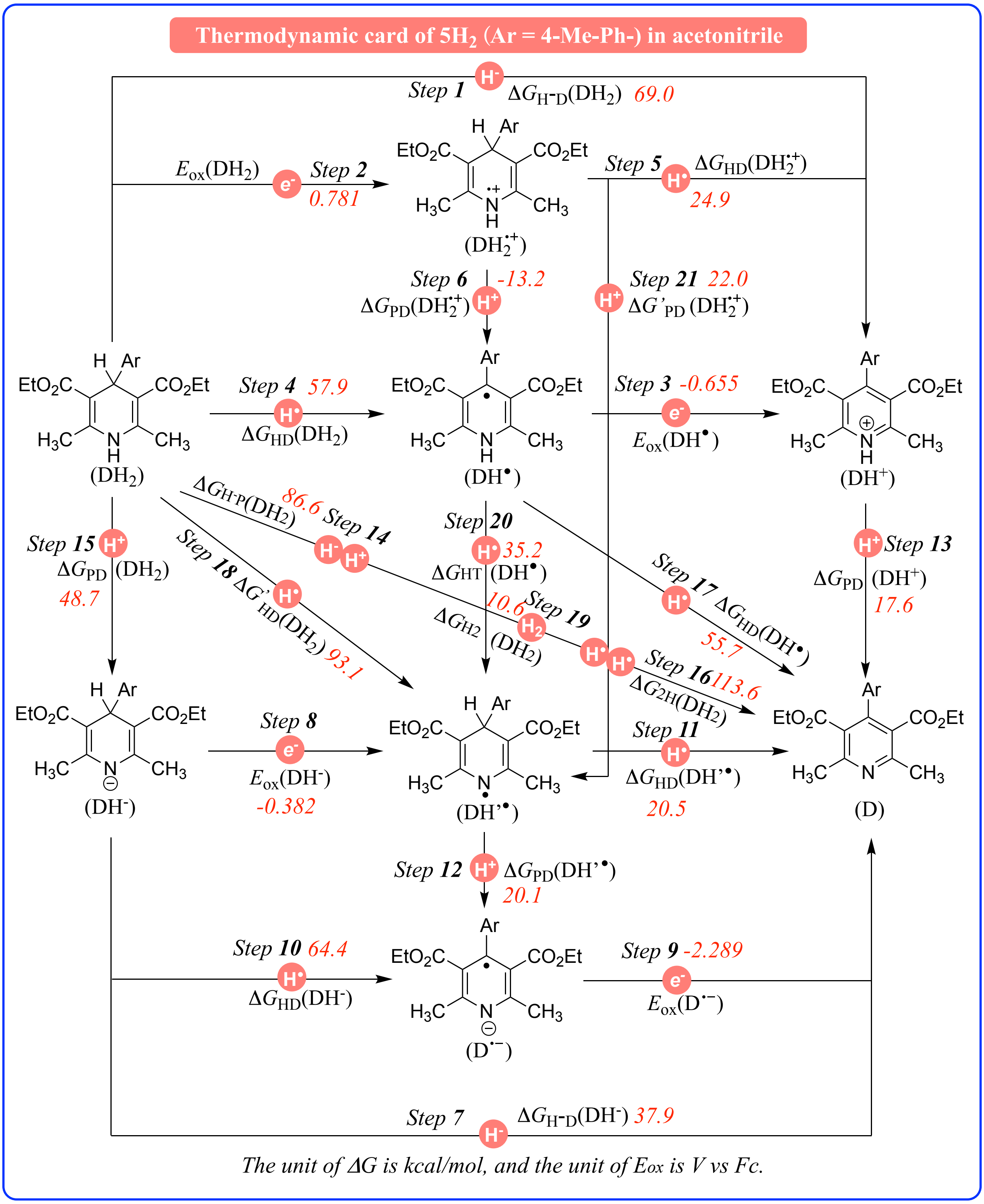

Figure S5. *Thermodynamic card of $5H_2$ in acetonitrile*

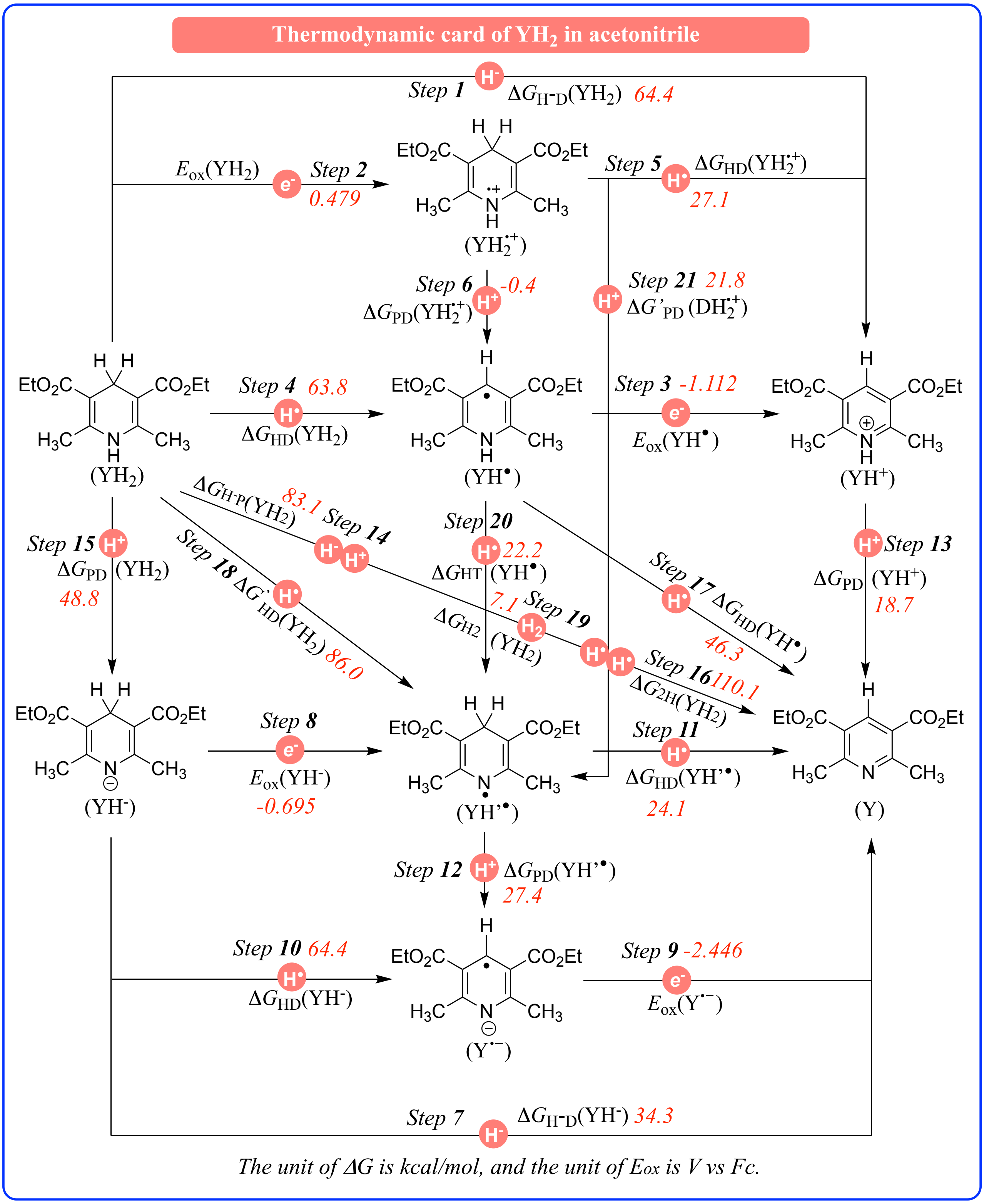

Figure S6. *Thermodynamic card of YH_2 in acetonitrile*
